# A pathogenic mechanism associated with myopathies and structural birth defects involves *TPM2*-directed myogenesis

**DOI:** 10.1172/jci.insight.152466

**Published:** 2022-06-22

**Authors:** Jennifer McAdow, Shuo Yang, Tiffany Ou, Gary Huang, Matthew B. Dobbs, Christina A. Gurnett, Michael J. Greenberg, Aaron N. Johnson

**Affiliations:** 1 Department of Developmental Biology, Washington University in St. Louis, St. Louis, Missouri, USA.; 2 Paley Orthopedic and Spine Institute, West Palm Beach, Florida, USA.; 3 Department of Neurology,; 4 Department of Orthopedic Surgery,; 5 Department of Pediatrics, and; 6 Department of Biochemistry and Molecular Biophysics, Washington University in St. Louis, St. Louis, Missouri, USA.

**Keywords:** Muscle Biology, Molecular genetics, Movement disorders, Muscle

## Abstract

Nemaline myopathy (NM) is the most common congenital myopathy, characterized by extreme weakness of the respiratory, limb, and facial muscles. Pathogenic variants in *Tropomyosin 2* (*TPM2*), which encodes a skeletal muscle–specific actin binding protein essential for sarcomere function, cause a spectrum of musculoskeletal disorders that include NM as well as cap myopathy, congenital fiber type disproportion, and distal arthrogryposis (DA). The in vivo pathomechanisms underlying *TPM2*-related disorders are unknown, so we expressed a series of dominant, pathogenic *TPM2* variants in Drosophila embryos and found 4 variants significantly affected muscle development and muscle function. Transient overexpression of the 4 variants also disrupted the morphogenesis of mouse myotubes in vitro and negatively affected zebrafish muscle development in vivo. We used transient overexpression assays in zebrafish to characterize 2 potentially novel *TPM2* variants and 1 recurring variant that we identified in patients with DA (V129A, E139K, A155T, respectively) and found these variants caused musculoskeletal defects similar to those of known pathogenic variants. The consistency of musculoskeletal phenotypes in our assays correlated with the severity of clinical phenotypes observed in our patients with DA, suggesting disrupted myogenesis is a potentially novel pathomechanism of *TPM2* disorders and that our myogenic assays can predict the clinical severity of *TPM2* variants.

## Graphical abstract

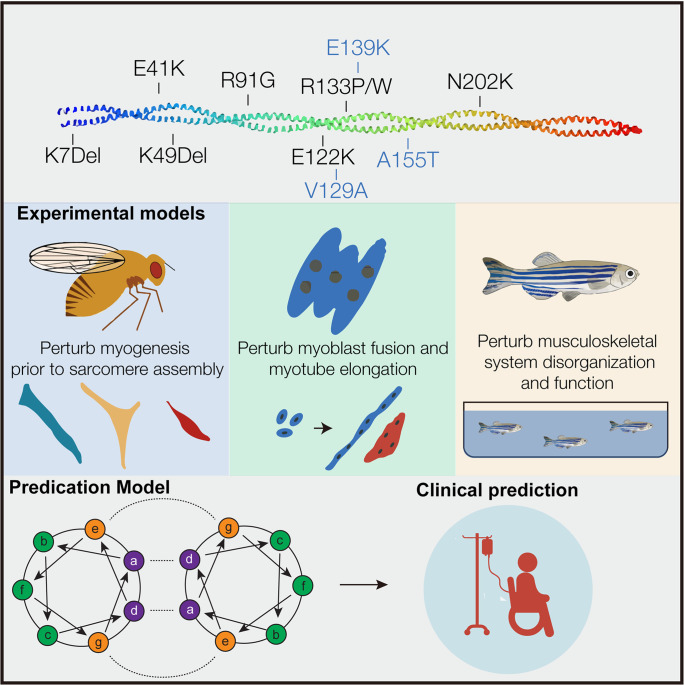

## Introduction

Tropomyosins are obligate actin binding proteins that form hetero- and homodimers (1). Head-to-tail tropomyosin polymers assemble along the length of actin filaments, and in the sarcomere tropomyosin regulates contractility by controlling the ability of thick filament myosin to access actin thin filaments (2). To initiate muscle contraction, Ca^2+^ released from the sarcoplasmic reticulum binds to sarcomeric troponin, which alters thin filament confirmation. The intermediate thin filament confirmation allows myosin to contact actin and further displace tropomyosin to drive maximal thin filament sliding and complete contraction (2).

Tropomyosin is encoded by 4 loci in humans (*TPM1*, *TPM2*, *TPM3*, and *TPM4*), with *TPM2* and *TPM3* being the predominant skeletal muscle isoforms (1). Pathogenic *TPM2* variants are causative of congenital skeletal muscle diseases, and much attention has been given toward understanding how *TPM2* variants disrupt sarcomere function. However, tropomyosin also functions outside of the sarcomere to regulate cytoskeletal changes that drive cell migration and cellular metastasis (3–5). Since skeletal muscle development depends on cytoskeletal dynamics to direct muscle precursor migration (6) and myofiber morphogenesis (7, 8), it is distinctly possible that *TPM2* variants adversely affect cytoskeletal dynamics prior to sarcomere assembly, which could disrupt overall muscle myogenesis.

Congenital diseases associated with *TPM2* include nemaline myopathy (NM) and cap myopathy (CM), which are both associated with extreme muscle weakness (hypotonia) (9–13). The diagnostic features for NM and CM are the presence of nemaline bodies and cap-like structures on muscle biopsy. Pathogenic *TPM2* variants are also causative of congenital fiber type disproportion (CFTD), in which highly oxidative type 1 myofibers are predominant and visibly hypotrophic (14). Patients with CFTD are also hypotonic.

A fourth congenital disease associated with *TPM2* is distal arthrogryposis (DA). The heterogeneity of DA clinical phenotypes has necessitated subtype classifications with hierarchical criteria (15). *TPM2* variants are causative of DA type 1 (DA1) (16), which is characterized by contractures of the hands and feet, including permanently bent fingers (camptodactyly) and clubfoot (talipes equinovarus) (15). *TPM2* variants are also associated with DA type 2B (DA2B) (17, 18), which is characterized by facial abnormalities in addition to contractures of the extremities (15). Patients with DA often show hypotonia (19, 20), suggesting skeletal muscle dysfunction contributes to the overall disease mechanism.

*TPM2* variants are also causative of Escobar variant of multiple pterygium syndrome (EVMPS) (19, 21, 22). Patients with EVMPS show joint contractures similar to those reported for patients with DA, but EVMPS is differentiated from DA by the presence of webbing (pterygia) at the neck, elbows, or knees (23). It is important to note that hypotonia often extends to the diaphragm in patients carrying *TPM2* variants, which may require lifelong respiratory intervention (13, 14, 19). The broad spectrum of clinical phenotypes associated with *TPM2* mutations has obscured a clear understanding as to how pathogenic *TPM2* variants disrupt skeletal muscle form and function.

**Figure 1:**
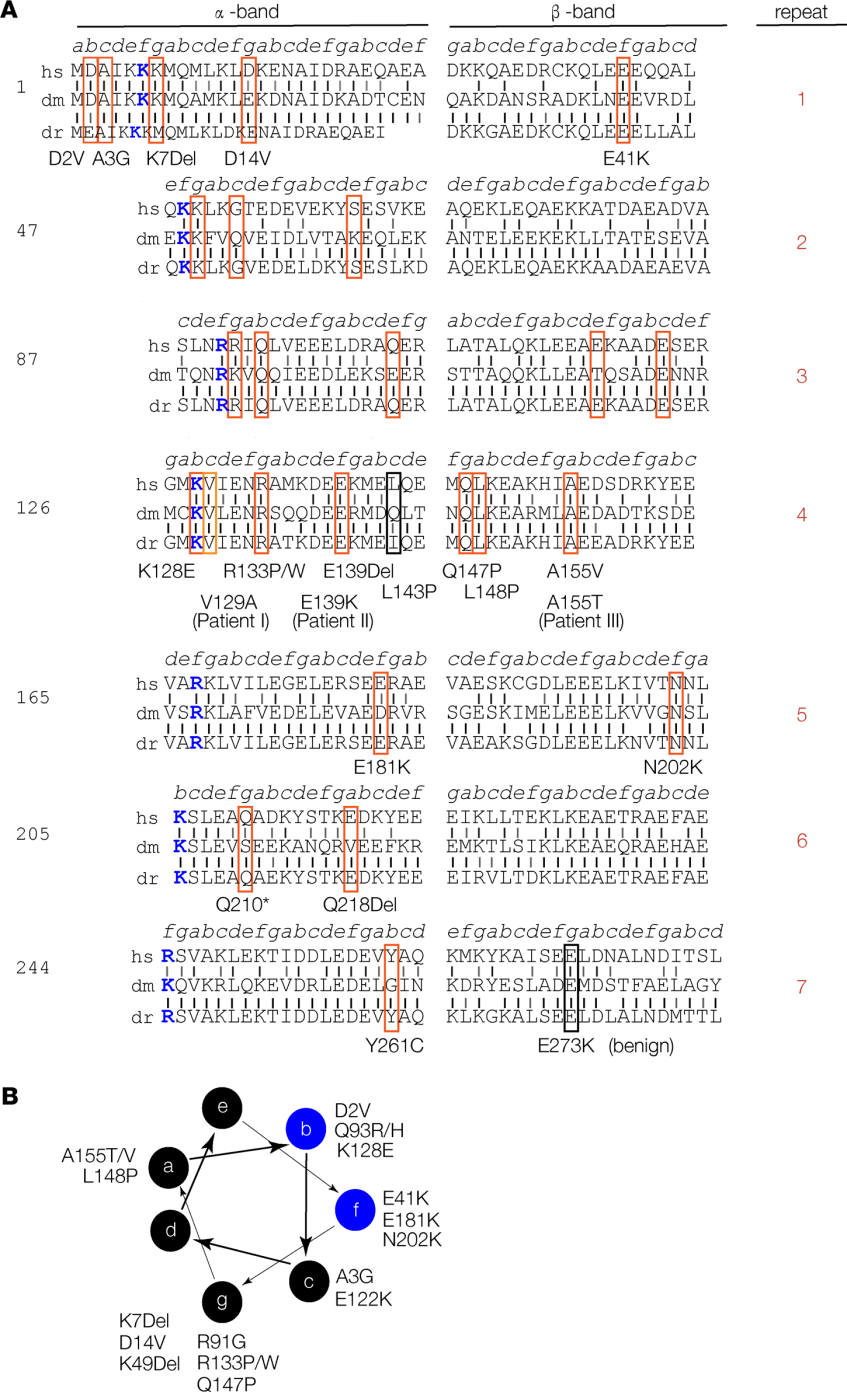
*TPM2* residues associated with pathogenic variants are conserved. (**A**) TPM2 conservation and sequence structure. TPM2 protein sequence divided into 7 quasi-repeats, split into α- and β-bands as described before (26). Actin-binding residues are colored blue. Vertical black lines show identical residues between the human (hs), Drosophila (dm), or zebrafish (dr) proteins; gray lines show similar residues. A total of 25 pathogenic *TPM2* coding region variants have been reported (19). We identified 2 potentially novel variants (V129A and E139K) and 1 recurring variant (A155T) in patients with musculoskeletal birth defects. Variants affecting conserved residues are boxed in red; nonconserved pathogenic variants are boxed in black. (**B**) Conserved pathogenic variants disproportionately cluster to a single topographical position. Diagram showing the 7–amino acid heptad (*a*–*g*) of the TPM2 coiled-coil, as described (62). A subset of *b* and *f* residues binds actin (blue circles). There are 7 conserved pathogenic variants mapped to residues in position *g*.

While the in vivo disease mechanisms that underlie *TPM2*-associated disorders are incompletely understood, the inheritance of *TPM2* congenital diseases follows an autosomal dominant pattern (9, 24). One notable exception is the pathogenic variant Q210*, which was shown to be autosomal recessive in a consanguineous family with EVMPS (25). A total of 30 pathogenic *TPM2* variants have been reported, and the variants themselves show a fairly even distribution along the protein (Figure 1A) (19). TPM2 is composed of 7 quasi-repeats, each divided into 1 α-sheet and 1 β-sheet, with 1 residue per quasi-repeat binding actin (26). In addition to the quasi-repeats, TPM2 forms a coiled-coil that follows the typical heptad repeat of 7 residues, labeled *a*–*g*, where *b* and *f* residues interact with actin and *g* residues are charged. Surprisingly, only 1 pathogenic variant changes an actin binding residue (K128E) (19), while 7 variants cluster to charged *g* positions (Figure 1B). The molecular genetics of *TPM2*-related disorders argues that pathogenic *TPM2* variants are dominant, gain-of-function mutations that indirectly disrupt tropomyosin-actin interactions.

Extensive biochemical studies have been used to understand the gain-of-function phenotypes *TPM2* variants induce. Thin filaments, or even entire muscle fibers, can be reconstituted in vitro to assay myosin-driven actin motility (26–32). Reconstituted thin filaments contain actin, tropomyosin, and troponin, such that actin motility can be measured in response to a Ca^2+^ gradient. Actin motility assays have shown that some *TPM2* variants increase Ca^2+^ sensitivity, causing maximum actin motility to be reached at comparatively low Ca^2+^ concentrations, while other variants reduce Ca^2+^ sensitivity (Table 1) (26, 32–34). The addition of fluorescence probes and proteins to actin motility assays reveals that the Ca^2+^ sensitivity of *TPM2* variants correlates with the ability of troponin and myosin to shift tropomyosin away from actin and that pathogenic substitutions alter tropomyosin flexibility (27–31). Since tropomyosin often exists as a heterodimer, *TPM2* variants likely act as gain-of-function mutations by altering Ca^2+^ sensitivity when dimerized with wild-type isoforms (28, 29, 35). Despite these extensive studies into the biochemical properties of *TPM2* mutations, pathogenic *TPM2* variants have rarely been characterized in vivo.

We set out to model *TPM2* congenital disorders in vivo, with the prediction that *TPM2* mutations would adversely affect muscle development and function. *TPM2* has been deleted in mice, and heterozygotes showed compromised lens regeneration (36). However, genome-edited *TPM2* variants have not been reported in any organism to our knowledge. A total of 28 pathogenic variants that affect the *TPM2* coding region have been reported, and we used transgenic overexpression in Drosophila, mammalian cell culture, and zebrafish embryos to study a representative collection of variants. Our studies revealed that pathogenic *TPM2* variants disrupt muscle development and muscle function. Transient overexpression proved to be a useful strategy for identifying *TPM2*-related disease mechanisms, so we used these assays to characterize 3 variants we identified in patients with musculoskeletal disorders. The variants V129A, E139K, and A155T caused phenotypes similar to those we identified for known pathogenic variants, providing additional evidence that the variants are in fact pathogenic. In addition, phenotypic consistency among our assays correlated with the severity of patient phenotypes, suggesting our disease models have the power to predict the clinical severity of *TPM2* variants. These studies identify defects in muscle development as a component for the etiology of *TPM2*-related disorders.

## Results

We set out to model *TPM2*-related diseases in vivo by characterizing a panel of variants that represent the key characteristics of the spectrum of *TPM2* pathogenic variants. A set of 8 variants in highly conserved residues are causative of the 5 *TPM2*-associated disorders, including NM (K7Del, E41K, R133W), CM (K49Del, N202K), CFTD (E122K, R133P), DA (K7Del, E41K, R91G, R133W), and EVMPS (R133W; Table 1). The representative variants are also equally distributed between α-sheets (K7Del, K49Del, R91G, R133P/W) and β-sheets (E41K, E122K, N202K; Figure 1A). With respect to the TPM2 coiled-coil heptad repeat, pathogenic variants generally cluster to *b*, *f*, and *g* residues (Figure 1B). The 8 representative variants clustered to the *f* (E41K, N202K) and *g* (K7Del, K49Del, R91G, E122K, R133P) positions, which is consistent with the overall distribution of pathogenic variants along the coiled-coil heptad (Figure 1B). The variants K7Del, E41K, K49Del, R91G, E122K, R133P, R133W, and N202K are thus a representative collection of *TPM2* mutations causative of congenital disease.

### TPM2 variants disrupt myogenesis.

**Figure 2:**
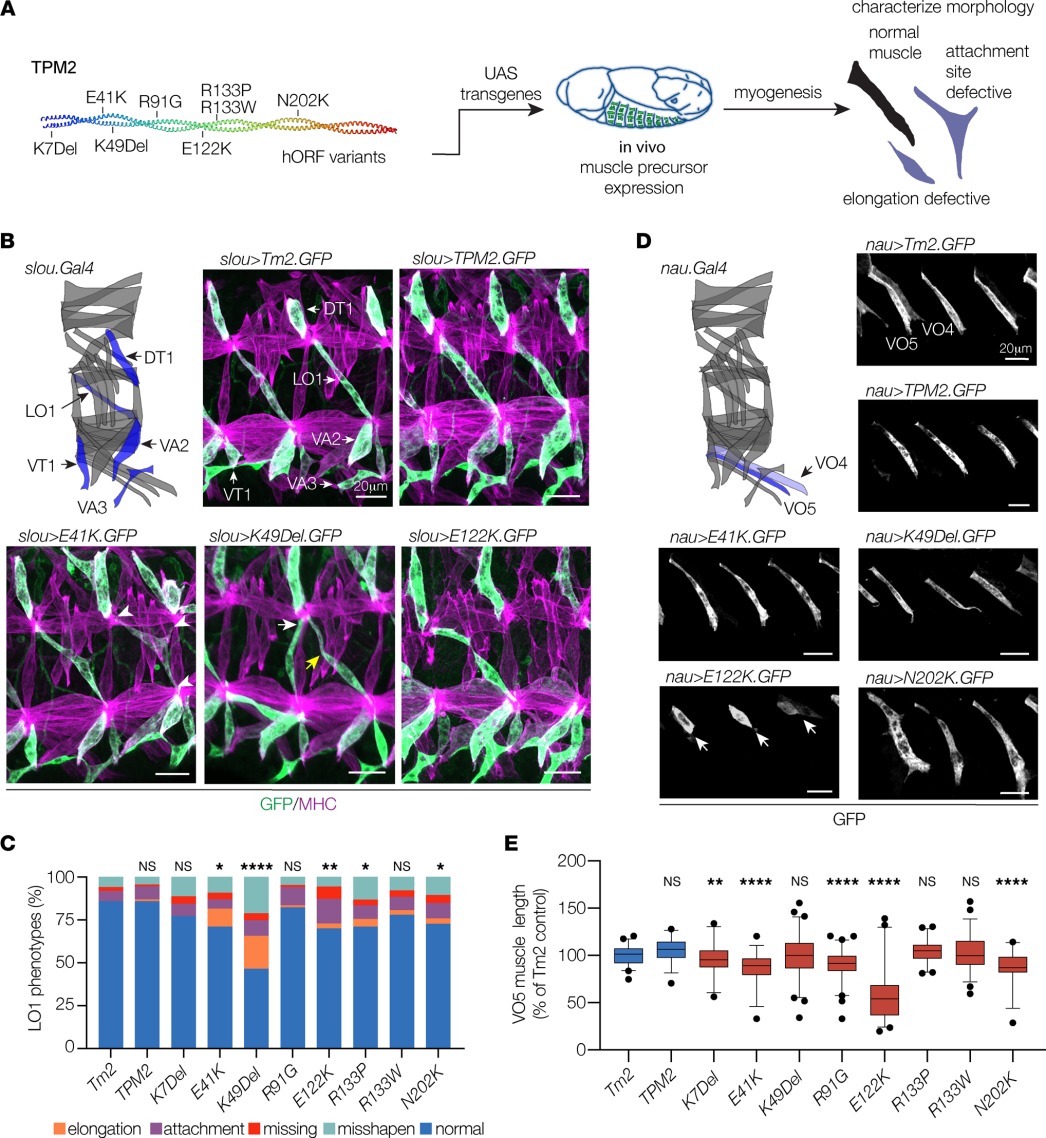
*TPM2* variants disrupt muscle development in Drosophila. (**A**) Transgenic expression assays used to characterize the effects of *TPM2* variants on myogenesis. (**B** and **C**) *TPM2* variants caused multiple phenotypes in *slou*-expressing muscles. (**B**) Diagram showing the 30 body wall muscles in an embryonic hemisegment; *slou.Gal4*-expressing muscles are shown in blue (modeled after ref. 7). Confocal micrographs of stage 16 embryos that expressed GFP-tagged Drosophila *Tropomyosin 2* (*Tm2*), wild-type human *TPM2*, or pathogenic *TPM2* variants (green), colabeled with myosin heavy chain (MHC, violet). Two hemisegments are shown for each embryo. Variant-expressing LO1 muscles showed multiple phenotypes, including rounded muscles (elongation), muscles attached to an incorrect tendon (wrong tendon, white arrows), muscles attached to 3 tendons (multiple tendons, white arrowheads), muscles absent from a segment (missing), and muscles with bent or hook-shaped morphology (misshapen; yellow arrows). (**C**) Histogram of variant phenotypes. (**D** and **E**) *TPM2* variants reduced muscle length in *nau*-expressing muscles. (**D**) The *nau.Gal4*-expressing muscles are diagramed in blue. Confocal micrographs of stage 16 embryos that expressed GFP-tagged transgenes, labeled for GFP. Variant-expressing VO5 muscles were short or rounded, but other parameters of muscle morphology were largely normal. E122K expressing muscles showed the strongest phenotype (white arrows). GFP expression in VO4 muscles was highly variable. (**E**) Box plot showing VO5 length normalized to *Tm2*-expressing control. Significance versus *Tm2*-expressing muscles was determined by Fisher’s exact test (**C**) or 1-way ANOVA (**E**). Error bars, standard error of the mean (SEM). *(*P* < 0.05), **(*P* < 0.01), ***(*P* < 0.001), ****(*P* < 0.0001). *n* ≥ 66 muscles per variant; minimum 9 embryos per variant. Scale bars, 20 μm.

Tropomyosin 2 (Tm2) is the Drosophila ortholog of TPM2, and the 2 proteins show a high degree of sequence conservation (Figure 1A). Overexpression studies in Drosophila have successfully modeled pathogenic variants in myosin heavy chain 3 (*MYH3*) associated with DA (37), so we used the binary UAS-GAL4 system to express GFP-tagged Drosophila *Tm2*, wild-type human *TPM2*, and the set of 8 *TPM2* variants in Drosophila embryonic muscle precursors. To minimize mRNA expression differences among the variants, UAS constructs were targeted to a common genomic landing site. The expression of endogenous Tm2, and the Gal4 drivers *slou.Gal4* and *nau.Gal4*, initiates immediately after muscle precursor specification (7, 8). In addition, *slou.Gal4* and *nau.Gal4* expression is spatially restricted to non-overlapping populations of muscle precursors (7). We used *slou.Gal4* and *nau.Gal4* to activate GFP-tagged transgenes in subpopulations of muscles and quantify muscle morphology at single-cell resolution (Figure 2A).

Embryonic muscles are named by their position and orientation in the segment, and the longitudinal oblique 1 (LO1) muscle shows a stereotypical oblique morphology (Figure 2B). LO1 muscles that expressed GFP-tagged *TPM2* variants under the control of *slou.Gal4* showed several abnormalities, including rounded and generally misshapen morphologies and attachments to the wrong tendon (Figure 2, B and C). Variant-expressing LO1 muscles also failed to develop in the correct position and were sometimes missing by the end of myogenesis (Figure 2, B and C). The frequency of LO1 muscle phenotypes was higher in muscles that expressed *TPM2* variants than in muscles that expressed wild-type *TPM2* or Drosophila *Tm2*, and LO1 muscles that expressed *K49Del* showed the highest frequency of muscle defects within the set of 8 pathogenic variants (Figure 2C). Ventral oblique 5 (VO5) muscles that expressed GFP-tagged pathogenic variants under the control of *nau.Gal4* were significantly shorter than VO5 muscles that expressed wild-type *TPM2* or *Tm2* (Figure 2, D and E). Among the variants tested, E122K caused the strongest phenotype in VO5 muscles (Figure 2E). Overall, our transgenic overexpression studies showed 7 of the 8 representative variants acted as gain-of-function mutations, demonstrating that pathogenic *TPM2* variants disrupt myogenesis in Drosophila.

Myogenesis is a multistep process that initiates with the specification of mononucleate muscle precursors known as myoblasts, which differentiate and fuse with each other to form multinucleate myotubes. Concurrent with myoblast fusion in Drosophila, nascent myotubes extend bilateral projections toward tendon cells at the segment border and identify correct muscle attachment sites through the process of myotube guidance. After attaching to tendon cells, myotubes assemble sarcomeres and mature into contractile myofibers (7). The phenotypes we identified in *TPM2*-expressing muscles occurred prior to sarcomere assembly, suggesting pathogenic *TPM2* variants could perturb myoblast fusion and myotube guidance prior to sarcomere assembly.

**Figure 3:**
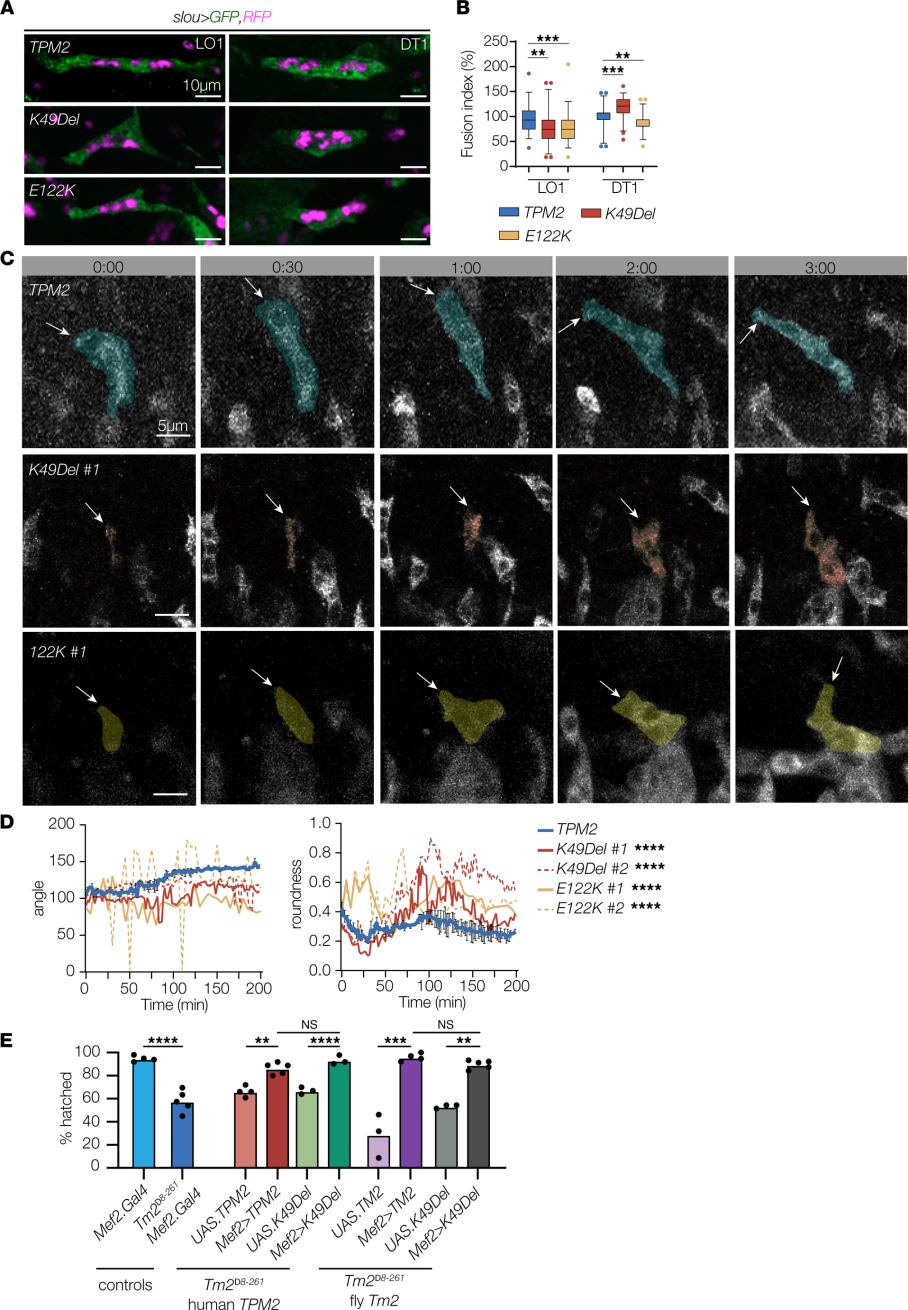
*TPM2* variants disrupt myoblast fusion and myotube guidance. (**A**) Myoblast fusion assays. Confocal micrographs of stage 16 embryos that expressed cytoplasmic EGFP (green), nuclear RFP (violet), and wild-type or variant *TPM2* under the control of *slou.Gal4*. DT1 and LO1 muscles that expressed K49Del or E122K showed a substantial change in the number of myonuclei compared with controls. The number of myonuclei in other *slou*-expressing muscles was unaffected. (**B**) Quantification of myoblast fusion. Fusion index indicates altered myoblast fusion in variant-expressing myotubes. *n* ≥ 45 myotubes per variant; minimum 8 embryos per variant. (**C**) Live imaging stills of LO1 myotubes in stage 12–15 embryos that expressed GFP-tagged TPM2. Transgene expression was controlled by *slou.Gal4*. Live imaging initiated when GFP fluorescence was first detected (0min). Dorsal leading edges (arrows) of control myotubes elongated to the dorsal anterior of the segment. Dorsal leading edges of variant-expressing myotubes failed to elongate or elongated toward the posterior of the segment. #:## (hr:min). (**D**) Quantification of myotube guidance. Control myotubes showed a stable myotube angle (consistent elongation toward a muscle attachment site) and a low roundness score (more linear). Variant-expressing myotubes showed fluctuating myotube angles and a high roundness score (more circular). (**E**) Hatching assays. *Tm2^Δ8-261^* homozygous embryos had significantly lower hatching rates than controls (blue bars). *Tm2^Δ8-261^* embryos that expressed human *TPM2* or Drosophila *Tm2* under the control of *Mef2.Gal4* showed significantly improved hatching rates compared with *Tm2^Δ8-261^* embryos (red and violet bars). *Tm2^Δ8-261^* embryos that expressed K49Del also showed a significant improvement in hatching rates (green and gray bars), but hatching rates were comparable between *Tm2^Δ8-261^* embryos that expressed wild-type or K49Del variants. Significance was determined by 1-way ANOVA (**B** and **E**) or unpaired, 1-tailed Student’s *t* test (**D**). Error bars, SEM. **(*P* < 0.01), ***(*P* < 0.001), ****(*P* < 0.0001). Scale bars, 10 μm (**A**), 5 μm (**C**).

To test this hypothesis, we carried out in-depth myoblast fusion and myotube guidance assays on 2 variants that produced the strongest phenotypes in our transgenic overexpression studies, K49Del and E122K. Using the *slou.Gal4*-expressing dorsal transverse 1 (DT1) and LO1 muscles as a model, we found variant-expressing muscles had a significant reduction in myoblast fusion, except K49Del-expressing DT1 muscles, which showed enhanced fusion (Figure 3, A and B). Live imaging of LO1 myotube guidance showed the dorsal leading edge elongated to the dorsal anterior of the hemisegment, giving the LO1 muscle its characteristic oblique morphology (Figure 3, C and D, and Supplemental Video 1; supplemental material available online with this article; https://doi.org/10.1172/jci.insight.152466DS1). LO1 myotubes that expressed K49Del or E122K showed several unusual behaviors. Some K49Del-expressing myotubes would initiate elongation, but the leading edge would retract and show a rounded muscle phenotype at the end of myogenesis (Supplemental Video 1 and Figure 3, C and D). In other examples, the leading edge in K49Del-expressing myotubes would elongate appropriately, but the lateral membrane would form an ectopic leading edge and elongate to a third tendon cell (Supplemental Video 2 and Figure 3, C and D). In E122K-expressing myotubes, the dorsal leading edge would also elongate incorrectly toward the medial or posterior regions of the segment (Supplemental Video 3 and Figure 3, C and D). The studies suggest that the myogenic phenotypes in variant-expressing embryos are due to defects in myoblast fusion and myotube guidance.

### TPM2 variants disrupt muscle function.

In some cases, muscle weakness in patients with *TPM2*-associated myopathies is restricted to proximal muscles in infancy but then progresses to include distal muscles in adulthood (11). To model TPM2 myopathies beyond embryonic development, we expressed Drosophila *Tm2* and human *TPM2* in the body wall muscles of larvae homozygous for the null allele *Tm2*^Δ8-261^. Larvae develop through 3 distinct growth stages (L1–L3), which are characterized by extensive muscle hypertrophy (38). Muscle morphology was largely normal in *Tm2*^Δ8-261^ homozygous embryos, owing to a maternal contribution of *Tm2* (8), but only 58% of *Tm2*^Δ8-261^ embryos hatched into larvae (Figure 3E). Hatching assays have been used to associate defects in myoblast fusion, sarcomere function, and neurotransmitter release with reduced muscle performance (39–41). We found that *Tm2*^Δ8-261^ embryos that broadly expressed *Tm2* or *TPM2* in the musculature hatched at significantly higher rates than controls, suggesting muscle function was restored in *Tm2*^Δ8-261^ embryos (Figure 3E). Surprisingly, the hatching rate was not significantly different between *Tm2* and *TPM2* rescued embryos, between *Tm2* and *Tm2.K49Del* embryos, or between *TPM2* and *TPM2.K49Del* rescued embryos (Figure 3E). These data argue that Tm2 and TPM2 are functionally equivalent, at least in the context of hatching assays, and that wild-type and K49Del variants can substitute for Tm2 in *Tm2*-null embryos. However, *Tm2*^Δ8-261^ rescued embryos did not develop beyond the L1 stage, independent of the transgene used for the rescue, suggesting endogenous Tm2 is required outside of the musculature for viability or that the level of transgenic Tm2 expression is functionally incompatible with endogenous expression levels.

**Figure 4:**
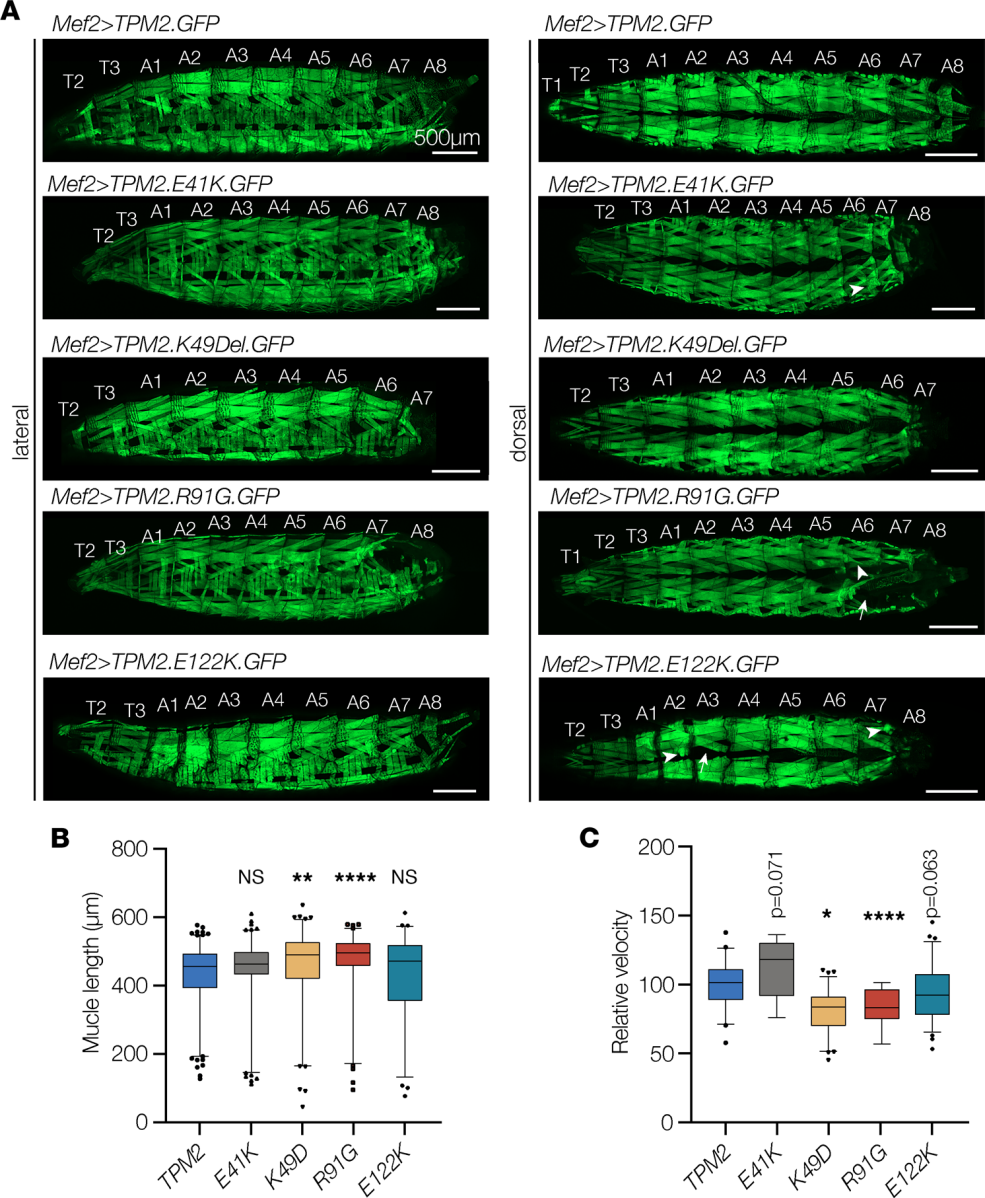
*TPM2* variants disrupt muscle function in Drosophila. (**A**–**C**) Larvae that expressed *TPM2* variants showed abnormal muscle morphology and impaired muscle function. (**A**) Confocal micrographs of live L3 larvae that expressed GFP-tagged *TPM2* or pathogenic *TPM2* variants (green) under the control of *Mef2.Gal4*. Larvae that expressed pathogenic variants often lacked muscles in segment A8. Muscles were also misshapen (arrowheads) or missing (arrow) in variant-expressing larvae. (**B**) Dorsal oblique muscle length (A2–A8). Muscles that expressed K49Del or R91G were significantly longer than controls. *n* ≥ 76 muscles per genotype. (**C**) Larval locomotion assays. Larvae that expressed K49Del or R91G were significantly slower than controls. *n* ≥ 9 larvae per genotype. Significance was determined by Kruskal-Wallis test. Error bars, SEM. *(*P* < 0.05), **(*P* < 0.01), ****(*P* < 0.0001). Scale bars, 500 μm.

To circumvent viability issues and model TPM2 variants in late larval stages, we broadly expressed K49Del and E122K throughout the musculature of otherwise wild-type larvae and assayed muscle morphology and function. K49Del-expressing muscles were significantly longer in L3 larvae than controls, which could reflect a reduced contractile state (Figure 4, A and B). Standardized larval locomotion assays have been developed to assess muscle function (42), and L3 larvae that expressed K49Del showed a significant reduction in locomotor activity compared with TPM2-expressing controls (Figure 4C). Larvae that expressed E122K did not show significant changes in muscle size or locomotion (Figure 4, B and C). To model additional representative variants in larvae, we extended our functional studies to include E41K and R91G (Figure 2, C and E). Larvae that expressed R91G showed significantly longer muscles and reduced locomotor activity, whereas larvae that expressed E41K did not show significant changes in muscle size or locomotion (Figure 4, A–C). All 4 variants caused muscle loss (Figure 4A), arguing voluntary crawling performance in L3 larvae is resistant to changes in the number of myofibers. In summary, larvae that expressed K49Del or R91G showed significant reductions in muscle function, while larvae that expressed E41K or E122K trended toward reduced muscle function. Our developmental and functional studies of known pathogenic variants in the fly show that no single variant caused significant phenotypes in every assay, suggesting multiple assays will be needed to evaluate the pathogenicity and relative severity of *TPM2* variants.

### TPM2 variants disrupt myotube morphogenesis in vitro.

**Figure 5:**
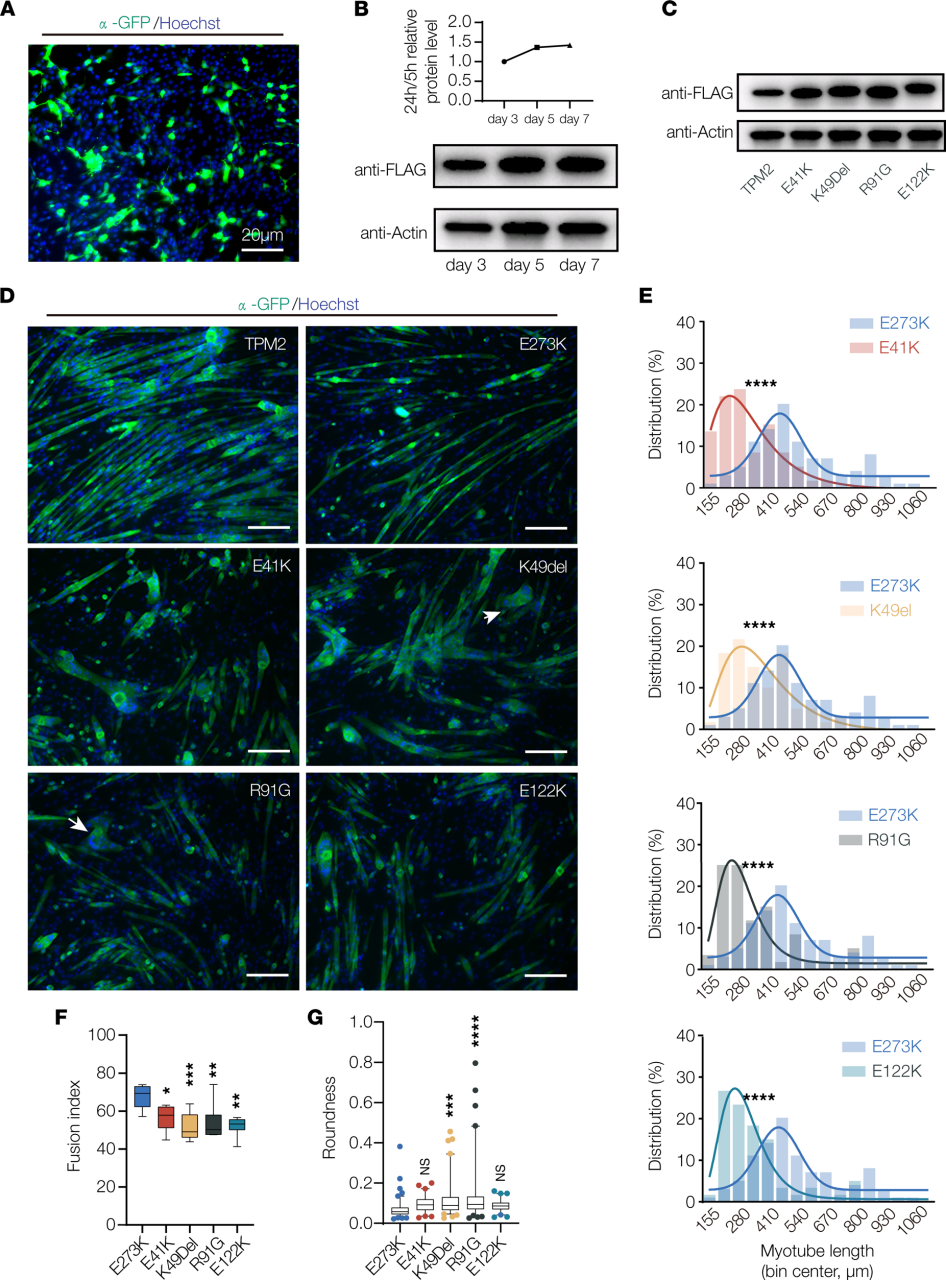
*TPM2* variants disrupt myotube morphogenesis. (**A**) Transfection efficiency. C2C12 myoblasts were transfected with TPM2.IRES.GFP and imaged 24 hours posttransfection. About 25% of cells were GFP positive. (**B**) Western blot of TPM2 expression. C2C12 cells were transfected with Flag-TPM2 and collected after 3, 5, and 7 days of differentiation. (**C**) Western blot of TPM2 variants. C2C12 cells were transfected with Flag-tagged variants and collected after 7 days of differentiation. Protein expression was similar among the variants. (**D**) C2C12 cells transfected with pathogenic *TPM2* variants showed impaired morphology. Confocal micrographs of cells fixed after 7 days in differentiation media and labeled for α-actinin (green) to detect differentiated myotubes and Hoechst to visualize myonuclei. Myotubes that expressed E41K, K49Del, R91G, and E122K appeared shorter than controls (wild-type TPM2 and the benign variant E273K). Variant-expressing myotubes were often rounded (arrows). Scale bars, 20 μm. (**E**) Myotube length distribution showing Gaussian distribution fit curves (solid lines). The length distribution of myotubes that expressed pathogenic variants skewed toward shorter lengths. (**F**) Quantification of myoblast fusion. Fusion index represents the number of nuclei in multinucleate myotubes; variant-expressing cells fused less than controls. (**G**) Roundness score. Individual myotubes were traced to calculate roundness; a score of 1.0 represents complete circularity. Myotubes that expressed K49Del and R91G were more round than controls. Significance was determined by unpaired, 1-tailed Student’s *t* test (**E**) or 1-way ANOVA (**F** and **G**). *n* ≥ 10 imaging fields per treatment. *(*P* < 0.05), **(*P* < 0.01), ***(*P* < 0.001), ****(*P* < 0.0001). Error bars, SEM.

To further evaluate the impact of *TPM2* variants on myogenesis, we used C2C12 cells to model muscle development in a vertebrate system. C2C12 cells are immortalized mouse myoblasts that fuse under differentiation conditions to form multinucleate myotubes capable of extensive elongation (43). K7Del, E41K, K49Del, and E122K have been studied in C2C12 cells, and while the variant proteins failed to localize correctly, the phenotypes of the variant expressing myotubes were not characterized (9, 44). We found that TPM2 expression constructs could be efficiently transfected in undifferentiated C2C12 myoblasts and that expression was maintained throughout differentiation (Figure 5, A and B, and uncut gels in the online supplemental material). In addition, endogenous *TPM2* mRNA was detectable in undifferentiated myoblasts, and expression increased 3.3-fold after 2 days of differentiation. We did not observe significant changes in the expression of wild-type or variant TPM2 proteins in differentiated myotubes (Figure 5C). C2C12 cells are thus a feasible model for studying the effects of *TPM2* variants on muscle development.

Myotubes that expressed wild-type *TPM2* were morphologically similar to control treated cells after 7 days of differentiation (Figure 5D). E273K (rs3180843, LOVD variant 0000446934) is a benign variant that was identified in a patient with normal muscle function. Using E273K as a secondary control, we found myotubes that expressed E273K were indistinguishable from myotubes that expressed wild-type *TPM2* (Figure 5, D–G). In contrast, myotubes that expressed E41K, K49Del, R91G, and E122K showed significant reductions in myoblast fusion and myotube elongation compared with E273K-expressing cells (Figure 5, D–F). K49Del- and R91G-expressing myotubes were also more circular than controls (Figure 5G), arguing these variants disrupted elongation to a greater extent than E41K and E122K. Since C2C12 cells develop independently of other musculoskeletal tissues, our results argue *TPM2*-related disease mechanisms act cell autonomously on developing myofibers. These studies also show that E273K can be used as a benign benchmark to evaluate variants of uncertain significance.

### TPM2 variants disrupt musculoskeletal system development in vivo.

**Figure 6:**
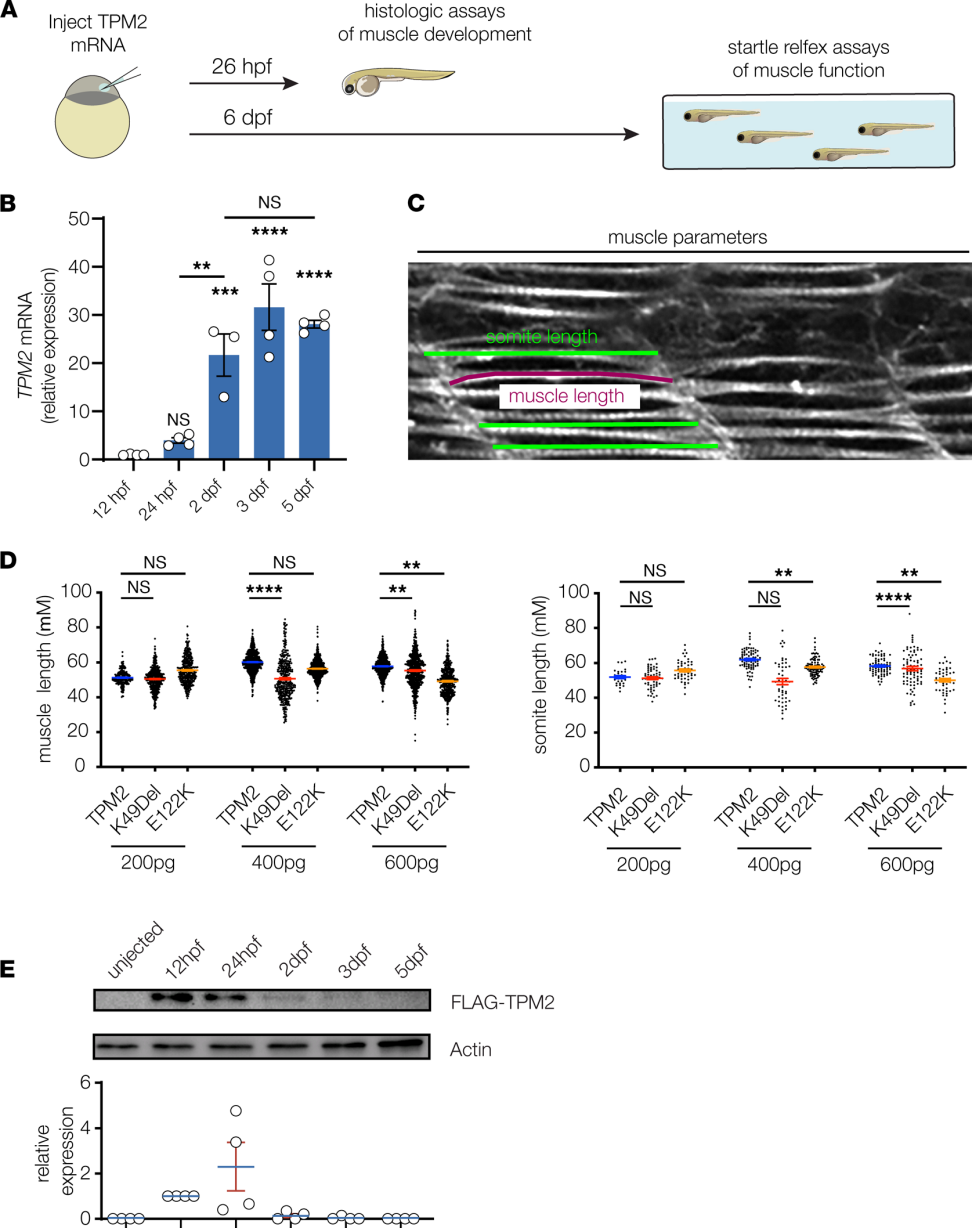
Transient overexpression assays in zebrafish. (**A**) Transient expression assays were used to characterize myogenic defects in zebrafish that expressed *TPM2* variants. One cell stage embryos were injected with control or variant-encoding mRNAs and raised under standard conditions to 26 hours postfertilization (hpf) for histological assays or to 6 days postfertilization (dpf) for locomotor assays. (**B**) Quantitative real-time PCR of zebrafish *TPM2* in wild-type embryos, normalized to 12 hpf. *n* = 4 larvae per sample; 3–4 replicates are shown. (**C**) Histologic measurements of 26 hpf larvae. Individual slow muscle fibers were traced in ImageJ (NIH) to determine muscle length (magenta line). Somite size was measured 3 times per somite and then averaged to calculate somite length. (**D**) Pathogenic *TPM2* variants caused dose-dependent defects in myofiber length and somite length. A gradient of mRNA doses were injected (200 pg, 400 pg, and 600 pg), and muscle morphology was assessed at each concentration. A dose of 600 pg produced consistent phenotypes in variant-expressing larvae but not in wild-type expressing larvae. Each data point represents an individual muscle fiber or somite. *n* ≥ 20 larvae per condition. (**E**) Western blot of injected TPM2. One-cell stage embryos were injected with 600 pg Flag-TPM2 mRNA, and lysates were collected at 12 hpf, 24 hpf, 2 dpf, 3 dpf, and 5 dpf. Robust TPM2 expression was detectable at 12 hpf and 24 hpf. Each data point graphed represents relative expression from 1 independent clutch. *n* ≥ 30 animals per sample; 4 replicates are shown. Significance was determined by unpaired, 1-tailed Student’s *t* test versus wild-type *TPM2*–injected fish. **(*P* < 0.01), ***(*P* < 0.001), ****(*P* < 0.0001). Error bars, SEM.

We recently modeled DA2A in zebrafish, and fish heterozygous for the knockin allele R672H in *MYH3* showed musculoskeletal abnormalities consistent with joint contractures (45). While the efficiency of genome-editing technologies in zebrafish is continuing to improve, the injection of variant-encoding capped mRNAs into fertilized embryos is a well-established tool for rapidly evaluating variant pathogenicity in developing embryos and larvae (46). Though this technique is difficult to use for large transcripts, we took advantage of the comparatively small *TPM2* coding sequence to generate and inject variant-encoding mRNAs into 1-cell stage embryos (Figure 6A). Similar to Drosophila, endogenous *TPM2* was detectable during myogenesis, and relative expression increased 3.9-fold from 12 to 24 hpf (Figure 6B). We injected a gradient of *TPM2* mRNA concentrations into 1-cell stage embryos and identified an optimized dose (600 pg) in which pathogenic variants produced musculoskeletal phenotypes while wild-type mRNAs did not (Figure 6, C and D). Protein from injected wild-type mRNA was strongly expressed 12–24 hpf and was nearly undetectable by 2 dpf (Figure 6E). However, clutch-to-clutch variation in protein expression at 24 hpf prevented us from assessing relative protein stability among the variants. Nonetheless, we successfully optimized mRNA injections and quantified musculoskeletal phenotypes to standardize and validate a transient overexpression assay in zebrafish.

**Figure 7:**
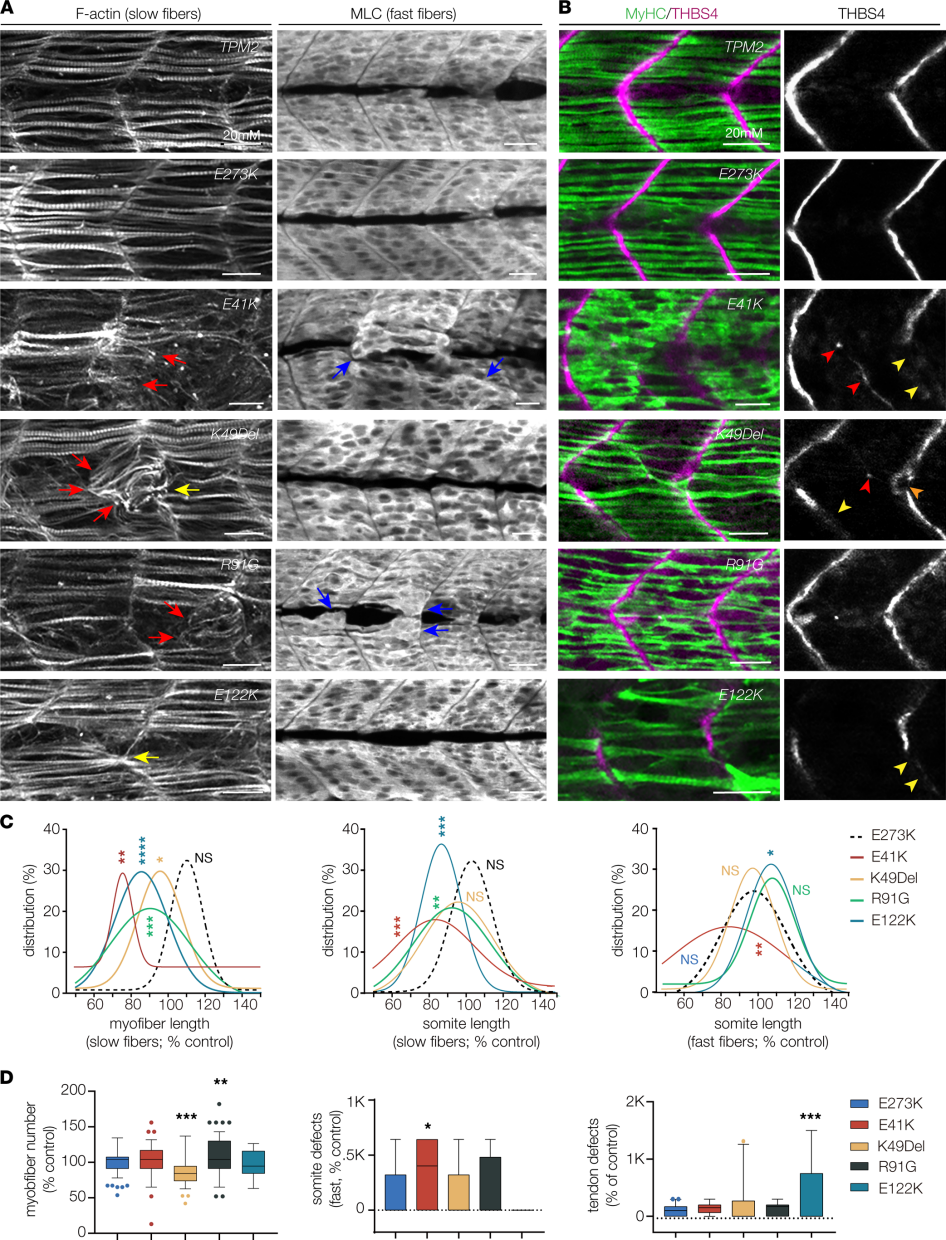
Pathogenic *TPM2* variants disrupt myogenesis in zebrafish. (**A**) Larvae that expressed pathogenic variants showed defects in muscle morphogenesis. Confocal micrographs of slow-twitch myofibers (left, F-actin) and fast-twitch myofibers (right, myosin light chain) in 26 hpf larvae injected at the 1-cell stage. Variant-expressing larvae showed multiple slow fiber phenotypes, including short fibers (red arrows) and fibers that clustered to a single attachment site (yellow arrowheads). Fast-twitch fiber morphology was largely normal in larvae that expressed pathogenic variants, although some larvae showed disorganized fast fibers (blue arrows). Larvae that expressed wild-type *TPM2* or the benign variant E273K had morphologically normal slow and fast fibers. (**B**) Larvae that expressed pathogenic variants showed defects in myosepta morphology. Confocal micrographs of 26 hpf larvae injected at the 1-cell stage, labeled for slow myofiber myosin heavy chain (MyHC, green) and the myosepta tendon marker Thrombospondin 4 (THBS4, violet). Variant-expressing larvae showed multiple phenotypes, including tendons that developed in the center of the somite (red arrowhead), bifurcated myosepta (orange arrowheads), and myosepta with broken thrombospondin expression (yellow arrowheads). The frequency of tendon phenotypes was substantial only for E122K. (**C**) Gaussian distribution fit curves. Length distributions in larvae that expressed pathogenic variants skewed toward shorter lengths for slow fibers but were less affected for fast fibers. *n* ≥ 48 somites per treatment. (**D**) Box plots showing slow fiber number and the frequency of morphology defects in fast muscle and myosepta. Larvae that expressed K49Del and R91G had significantly fewer slow fibers than larvae that expressed E273K. Morphology defects were restricted to E41K-expressing larvae (fast fibers) and E122K-expressing larvae (myosepta). *n* ≥ 11 larvae per treatment. Scale bars, 20 μm. Significance was determined by unpaired, 1-tailed Students *t* test (**C**) and 1-way ANOVA (**D**). *(*P* < 0.05), **(*P* < 0.01), ***(*P* < 0.001), ****(*P* < 0.0001). Error bars, SEM.

Slow- and fast-twitch myofibers are spatially distinct in zebrafish; slow fibers are found just beneath the epidermis and are superficial to the fast fibers. At 26 hpf, larvae that expressed wild-type or E273K *TPM2* showed normal musculoskeletal features, whereas larvae that expressed E41K, K49Del, R91G, and E122K had significantly shorter slow fibers than controls (Figure 7, A and C). In addition, the slow fibers were disorganized, and the myofiber ends often clustered at the somite boundary or at the center of the somite (Figure 7A). Somite length at the level of slow fibers was shorter in larvae that expressed E41K, R91G, and E122K compared with E273K controls, and fewer slow fibers were present in larvae that expressed K49Del and R91G (Figure 7, C and D). Somite length at the level of fast fibers was significantly changed in larvae that expressed E41K and E122K compared with E273K controls, and although fast fiber morphology was difficult to visualize at the single-cell level, larvae that expressed E41K showed defects in fast fiber organization (Figure 7, C and D).

Myofibers in the trunk attach to tendons in the myosepta, which are located along the somite boundaries and strongly express the tendon structural protein THBS4 (Figure 7B). Larvae that expressed wild-type or E273K showed a normal tendon pattern, whereas larvae that expressed K49Del and E122K showed multiple tendon phenotypes that included tendons incorrectly positioned in the center of the somite, bifurcated myosepta, and myosepta with broken or incomplete expression of thrombospondin (Figure 7, B and D). These in vivo studies provide further evidence that pathogenic *TPM2* variants disrupt musculoskeletal system development. In addition, the musculoskeletal phenotypes in our transient overexpression assay can faithfully distinguish benign *TPM2* variants from pathogenic variants.

### TPM2 variants disrupt muscle performance in vivo.

**Figure 8:**
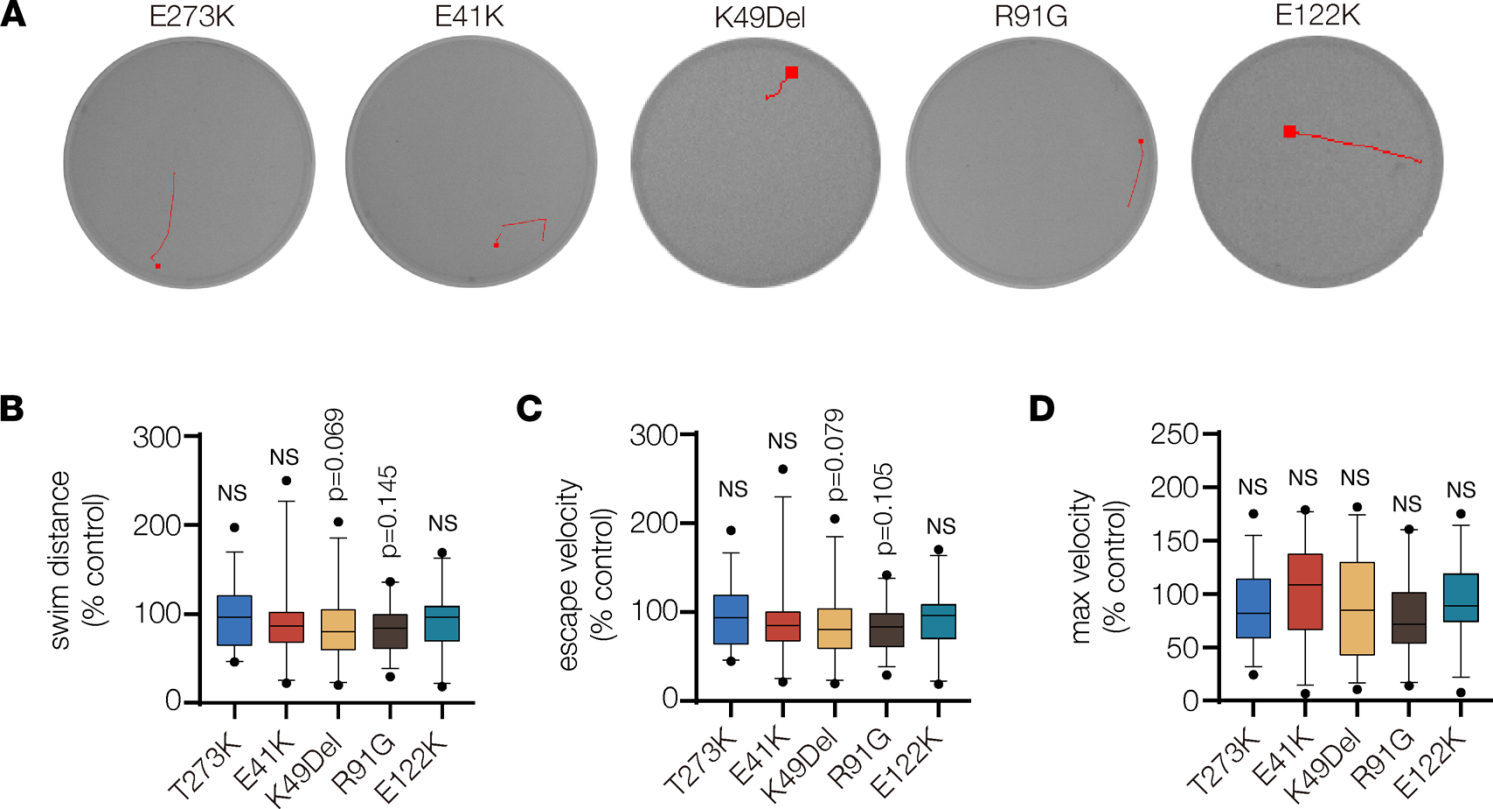
*TPM2* variants did not affect swim function. (**A**) Automated tracking of the startle response in 6 dpf larvae. EthoVision imaging software (Noldus) tracked larvae for 3 seconds after a mechanical stimulus. Red lines show the locomotor path of representative larvae. Endpoints are marked by a square. (**B**–**D**) Box plots showing swim function. The startle response in larvae that expressed E41K, K49Del, R91G, and E122K was not significantly different from larvae that expressed the benign variant E273K. Swim distance, average escape velocity, and maximum velocity of larvae that expressed *TPM2* variants were reported by imaging software and normalized to larvae that expressed wild-type *TPM2*. Significance was determined by 1-way ANOVA. *n* ≥ 25 larvae per treatment. Error bars, SEM.

The startle response in zebrafish larvae is a well-characterized reflex used to assay motor function, and we previously showed muscle function is compromised in a *MYH3* model of DA using the larval startle response (45). To understand if *TPM2* variants affect muscle function in zebrafish, we injected mRNAs into 1-cell stage embryos and ran automated tracking assays in larvae at 6 dpf (Figure 6A and Figure 8A). After a stimulus, the startle response induces a reflexive swim behavior that is quantified by distance swum, average escape velocity, and maximum velocity. Although transient overexpression of TPM2 variant mRNAs did not induce protein expression beyond 2 dpf (Figure 6E), we hypothesized that the developmental defects we observed in variant-expressing larvae would affect swim function at 6 dpf. Surprisingly, larvae that expressed pathogenic variants did not show significant changes in swim function compared to E273K-expressing larvae, although K49Del- and R91G-expressing animals trended toward reduced swim distance and escape velocity (Figure 8, B–D). One explanation for normal swim function in variant-expressing larvae is that muscle development continues beyond transient overexpression, which ends at 2 dpf. It is possible that newly developed and presumably wild-type muscle partially compensates for the developmental defects we identified at 26 hpf.

### TPM2 variants are identified in patients with musculoskeletal disorders.

**Figure 9:**
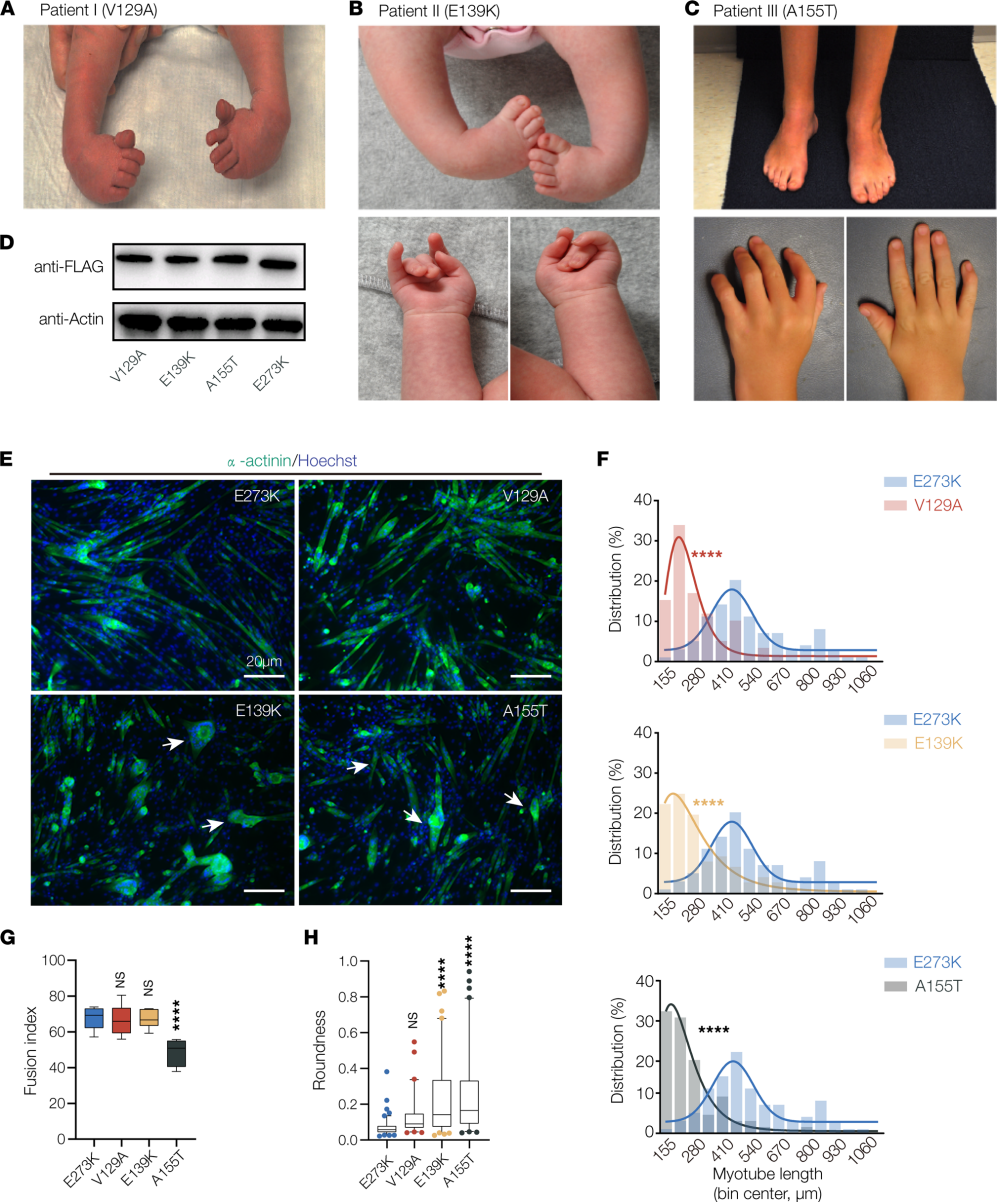
*TPM2* variants identified in patients with musculoskeletal disorders disrupt myotube morphogenesis. (**A**) Clinical features of patient I, diagnosed with bilateral clubfoot (shown here before treatment). (**B** and **C**) Clinical features of patient II and patient III, diagnosed with DA1. Photos for patient II show clubfoot before treatment, and photos of patient III show bilateral clubfoot after treatment. Individual III also developed lower extremity weakness as a child. Patient I and patient II are heterozygous for the novel variants V129A and E139K; patient III is heterozygous for the recurring variant A155T. (**D**) Western blot of TPM2 variants. C2C12 cells were transfected with Flag-tagged variants and collected after 7 days of differentiation. Protein expression was similar among V129A, E139K, and A155T. (**E**) C2C12 cells transfected with pathogenic *TPM2* variants showed defective morphology. Confocal micrographs of cells fixed after 7 days in differentiation media and labeled for α-actinin (green) to detect differentiated myotubes and Hoechst to visualize myonuclei. Myotubes that expressed V129A, E139K, and A155T appeared shorter than controls expressing the benign variant E273K. Variant-expressing myotubes were often rounded (arrows). Scale bars, 20 μm. (**F**) Myotube length distribution showing Gaussian distribution fit curves (solid lines). The length distribution of myotubes that expressed V129A, E139K, and A155T skewed toward shorter lengths. (**G**) Quantification of myoblast fusion. Fusion index represents the number of nuclei in multinucleate myotubes; cells that expressed A155T fused less than controls. (**H**) Roundness score. Individual myotubes were traced to calculate roundness; a score of 1.0 represents complete circularity. Myotubes that expressed E139K and A155T were more round than controls. Significance was determined by unpaired, 1-tailed Student’s *t* test (**E**) or 1-way ANOVA (**F** and **G**). *n* ≥ 10 imaging fields per treatment. ****(*P* < 0.0001). Error bars, SEM.

As part of our ongoing clinical sequencing of patients with musculoskeletal disorders, we identified 2 potentially novel *TPM2* variants and 1 recurring variant. Patient I presented with isolated bilateral clubfoot but no hand contractures and was found to be heterozygous for V129A (Figure 9A). Patients II and III are unrelated patients who were diagnosed with DA1, and were found to be heterozygous for E139K and A155T, respectively (Figure 9, B and C). Patient III also had mild distal lower extremity weakness and fatigue upon running. All 3 patients showed symptoms at birth, but none required interventions for motility or respiration (Table 2). There was no family history of arthrogryposis or clubfoot for any of the 3 patients, but parents and other family members were unavailable for genotyping. Two of the *TPM2* variants, V129A and E139K, had not previously been identified in patients with myopathies or arthrogryposes, although CM has been linked to the variant E139Del (12, 47). The third variant, A155T, was previously identified in a Chinese family with DA1 (48).

### Variants disrupt myotube morphogenesis in vitro.

To evaluate the pathogenicity of V129A and E139K, and to characterize the severity of A155T relative to other variants, we assayed the impact of each variant on C2C12 cell morphology. Similar to other TPM2 variants, there was no difference in the expression of V129A, E139K, and A155T proteins in differentiated myotubes compared to controls (Figure 9D). Myotubes that expressed V129A, E139K, and A155T showed reduced myotube elongation compared with E273K-expressing cells (Figure 9, E and F), and myotubes that expressed A155T showed reduced myoblast fusion (Figure 9G). E139K- and A155T-expressing myotubes were also more circular than controls (Figure 9H), arguing these variants disrupted elongation to a greater extent than V129A. Thus, myotubes that expressed V129A, E139K, and A155T showed more significant phenotypes than the benign variant E273K, which provides additional evidence that V129A, E139K, and A155T are pathogenic.

### TPM2 variants disrupt muscle development and function in zebrafish.

**Figure 10:**
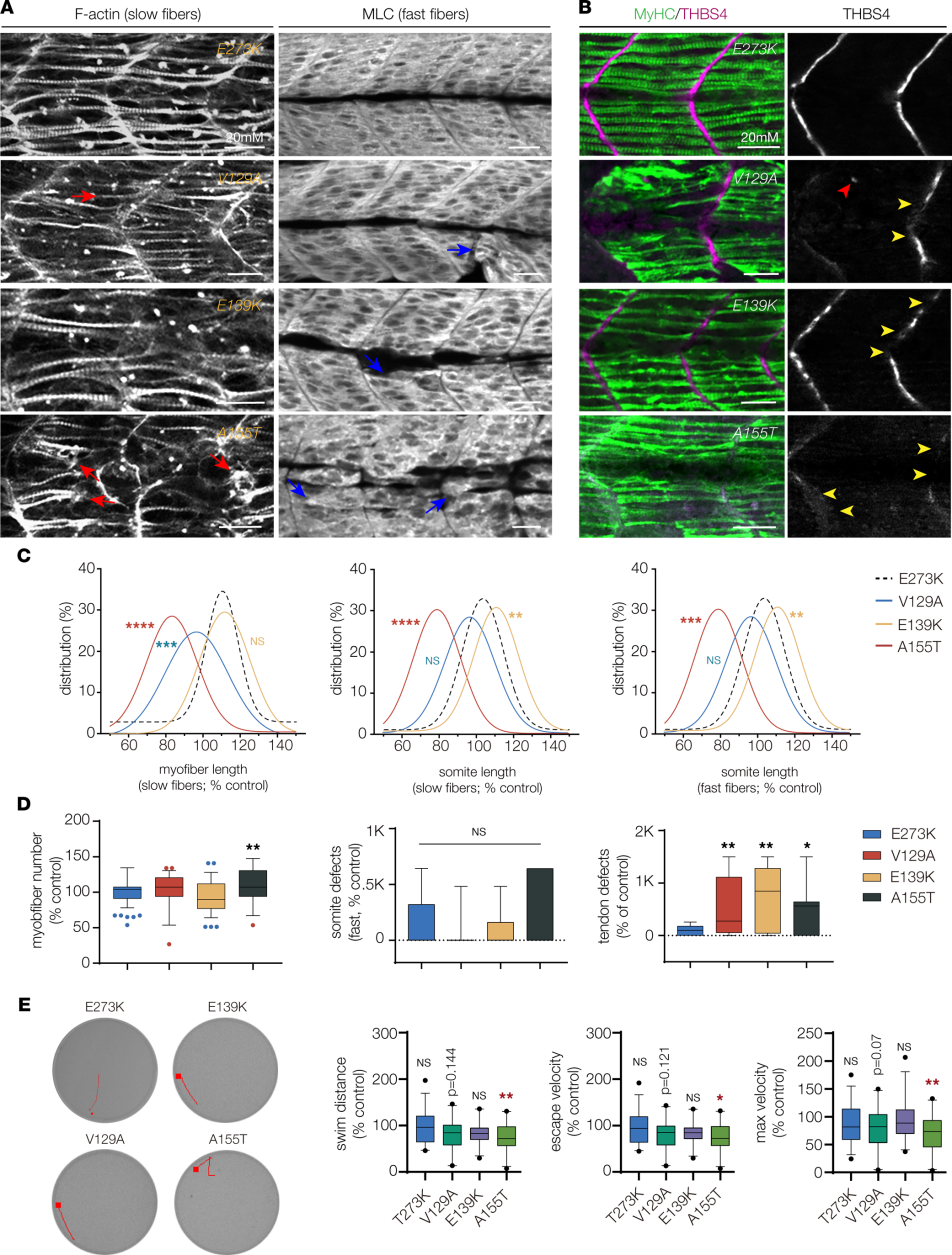
*TPM2* variants disrupt myogenesis and muscle function. (**A**) Larvae that expressed V129A, E139K, and A155T showed defects in muscle morphogenesis. Confocal micrographs of slow-twitch myofibers (left, F-actin) and fast-twitch myofibers (right, myosin light chain) in 26 hpf larvae injected at the 1-cell stage. Variant-expressing larvae had phenotypes including short slow fibers (red arrows) that often clustered at the center of the somite and disorganized fast fibers (blue arrows). Larvae that expressed wild-type *TPM2* or the benign variant E273K had morphologically normal myofibers. (**B**) Larvae that expressed V129A, E139K, and A155T showed defects in myosepta morphology. Confocal micrographs of 26 hpf larvae injected with *TPM2* RNAs at the 1-cell stage, labeled for slow myofiber myosin heavy chain (MyHC, green) and the myosepta tendon marker Thrombospondin 4 (THBS4, violet). Larvae that expressed pathogenic variants developed tendons in the center of the somite (red arrowhead) and showed broken thrombospondin expression (yellow arrowheads). (**C**) Gaussian distribution fit curves. Slow fiber length distributions in larvae that expressed V129A and A155T skewed toward shorter lengths. Somite size was smaller in larvae that expressed A155T and longer in larvae that expressed E139K. *n* ≥ 48 somites per treatment. (**D**) Box plots quantifying myofiber number and morphology defects. Larvae that expressed A155T had significantly fewer slow fibers than larvae that expressed E273K. Morphology defects were restricted to myosepta in larvae that expressed V129A, E139K, and A155T. *n* ≥ 7 larvae per treatment. (**E**) Automated tracking of the startle response in 6 dpf larvae, as described in Figure 8. Larvae that expressed A155T had reduced startle responses compared with E273K larvae. *n* ≥ 24 larvae per treatment. Swim parameters were normalized to larvae that expressed wild-type *TPM2*. Significance was determined by unpaired, 1-tailed Student’s *t* test (**C**) and 1-way ANOVA (**D** and **E**). *(*P* < 0.05), **(*P* < 0.01), ***(*P* < 0.001), ****(*P* < 0.0001). Error bars, SEM.

The residues affected by V129A, E139K, and A155T are conserved in zebrafish (Figure 1A), so we used our transient overexpression assay to further characterize the effect of each variant on musculoskeletal system development in vivo. Larvae that expressed V129A, E139K, and A155T showed defects in musculoskeletal morphogenesis similar to those that expressed known pathogenic variants (Figure 8, A and B). Slow fibers from larvae that expressed V129A and A155T were significantly shorter than in E273K-expressing controls (Figure 10, A and C). Variant-expressing slow fibers were also disorganized and often clustered to the center of the somite (Figure 10A). Somite length at the level of slow fibers was shorter in larvae that expressed A155T and longer in larvae that expressed E139K (Figure 10C). Fewer slow fibers were present in larvae that expressed A155T (Figure 10D). Somite length at the level of fast fibers was significantly changed in larvae that expressed V129A and A155T compared with E273K controls, but fast fiber morphology was unaffected (Figure 10, C and D). In addition, larvae that expressed V129A, E139K, and A155T showed a significant increase in the frequency of tendon phenotypes compared with E273K controls (Figure 10D).

We performed startle response assays on larvae that expressed V129A, E139K, and A155T and found A155T-expressing larvae showed significantly reduced swim distance, average escape velocity, and maximum velocity compared with E273K-expressing larvae (Figure 10E). V129A-expressing larvae trended toward reduced swim performance but were not significantly different from controls (Figure 10E). The *TPM2* variants we identified in patients with musculoskeletal disorders caused defects in musculoskeletal system development. In the case of A155T, functional defects persisted for 4 days longer than protein expression, suggesting the developmental phenotypes in larvae that expressed A155T were more severe than in larvae expressing any other variant. Our assays in C2C12 cells and zebrafish provided additional evidence that the potentially novel *TPM2* variants V129A and E139K are pathogenic, and the results argue that A155T is causative of *TPM2*-related musculoskeletal disorders.

## Discussion

To date, over 30 *TPM2* variants have been identified in patients with myopathies and arthrogryposes, but the biochemical properties of only a few variants have been tested. Furthermore, in vivo studies investigating the physiological consequences of pathogenic *TPM2* variants were largely lacking. We expressed *TPM2* variants in multiple model systems and found pathogenic variants disrupted muscle development and muscle function. By focusing on 4 pathogenic variants, we developed a transient overexpression assay in zebrafish that benchmarked musculoskeletal phenotypes of pathogenic variants against a known benign variant. Clinical sequencing of patients with structural birth defects identified 2 potentially novel *TPM2* variants, V129A and E139K, and a recurring variant, A155T, which we tested with our transient overexpression assay. V129A, E139K, and A155T caused musculoskeletal defects similar to those of the known pathogenic variants, and our analyses provide support for pathogenicity of all 3 variants. A155T produced the most consistent phenotypes of the variants we tested, and the clinical symptoms of the patient with the A155T variant were the most severe among the patients in our study. Our results argue that one pathomechanism of *TPM2*-related disorders is disrupted muscle development and that transient overexpression assays can efficiently characterize variants of uncertain significance identified in patients with musculoskeletal disorders.

Investigations of *TPM2*-related disease mechanisms have largely focused on understanding the role of TPM2 in the sarcomere. Thin filament motility assays have uncovered the biochemical properties of TPM2 variants in response to Ca^2+^, and the pathogenic variants tested so far have shown both increased and reduced Ca^2+^ sensitivity (Table 1). The basis for Ca^2+^ sensitivity is thought to reside in the flexibility or rigidity of the TPM2 dimer, which correlates with the ability of troponin and myosin to shift tropomyosin away from actin (27–31). Here, we found that *TPM2* variants disrupted muscle morphogenesis prior to sarcomere assembly in vivo. Our live imaging in *Drosophila* embryos showed myotubes that expressed K49Del and E122K had elongation defects and used inappropriate muscle attachment sites to adhere to the exoskeleton (Supplemental Videos 1–3). We observed similar myotube elongation defects in C2C12 cells that expressed pathogenic *TPM2* variants. Since C2C12 cells develop in the absence of positional cues from other tissues, our studies argue *TPM2* disease mechanisms act cell autonomously to disrupt myofiber morphogenesis prior to sarcomere assembly.

Tropomyosin has well-documented roles outside of the sarcomere in nonmuscle cells, most notably during cell migration. Dynamic changes to the cytoskeleton, coupled with changes in the expression of cell adhesion proteins, drive cell migration. Tropomyosins regulate the rate of actin polymerization and depolymerization (5, 49, 50), so it is not surprising that TPM2 and TPM3 are required for single-cell as well collective cell migration (3, 4). In vertebrates, myoblasts specified in somites migrate to sites of muscle morphogenesis, where they fuse to form myotubes that in turn elongate and attach to tenocytes (51, 52). During zebrafish myogenesis, myoblasts that give rise to slow- and fast-twitch myofibers are developmentally distinct. Slow myoblasts known as adaxial cells are specified medially, nearest the notochord, and migrate radially to form elongated myotubes on the superficial, outermost region of the somite (53). The slow-twitch region of the myotome in larvae that expressed pathogenic *TPM2* variants (Figure 7A and Figure 10A) bore a striking resemblance to the slow-twitch myotome of larvae with defective adaxial cell migration (54). It is possible that pathogenic *TPM2* variants disrupt myoblast cell migration in zebrafish, suggesting myoblast migration may be affected in patients with *TPM2*-related disorders.

Drosophila embryonic myoblasts do not migrate because muscles are specified at the site of myogenesis. However, similar to vertebrates, Drosophila myoblasts fuse to form myotubes, and all the *TPM2* variants we tested disrupted myoblast fusion (Figure 3, A and B, Figure 5F, and Figure 9G). Myotubes also elongate and identify muscle attachment sites (7). Myotube elongation and attachment site selection are collectively known as myotube guidance, which is similar to axon guidance in many respects. Myoblast fusion and myotube guidance depend on regulated changes to the actin cytoskeleton (7, 8, 55). The *TPM2* muscle morphogenesis defects we characterized in Drosophila, zebrafish, and cultured cells are likely the result of improperly regulated actin dynamics during myoblast migration, myoblast fusion, and myotube elongation. DA and amyoplasia (absence of muscle) were often thought to be distinct clinical diagnoses, but amyoplasia was recently reported in a case of congenital DA (56). Our studies provide additional support for a model in which *TPM2* variants disrupt muscle development.

At present, 14 *TPM2* variants have been identified in patients with myopathies and arthrogryposes in which the significance of the variant has not been definitively defined (Table 3). The number of *TPM2* variants with uncertain significance is likely to increase because these variants are being identified in patients with isolated clubfoot, which is much more common than myopathies or arthrogryposes. The incidence of clubfoot in the United States is 1:1000 live births, but the underlying causes are often unknown. One approach toward understanding isolated clubfoot is to expand clinical sequencing, which will likely uncover novel *TPM2* variants. Phenotypic variability among patients with *TPM2* variants can make genotype-phenotype correlations difficult (19), but the stringency of our benchmarked assays unambiguously defined variants as pathogenic or benign.

Transient overexpression in zebrafish has several advantages over the assays we developed. First, variant mRNAs can be generated from any vector with a T3, T7, or Sp6 promoter without requiring specialized expression or transgenic vectors. Second, mRNA injections into 1-cell stage embryos are fast, and the assays are generally complete and analyzed statistically within 2–3 days. Several weeks of breeding are required to identify and characterize stable transgenes in Drosophila, and C2C12 cell assays require 7 days of differentiation after transfection. In addition to speed and convenience, zebrafish carry out all stages of myogenesis and generate a musculoskeletal system with an endoskeleton. Zebrafish are therefore more similar to patients than our other models. Considering the advantages of zebrafish as a system, our transient overexpression assay could be further used to uncover new pathogenic variants of *TPM2* and to test additional genes of uncertain significance that contribute to the pathology of isolated clubfoot or other musculoskeletal disorders.

**Figure 11:**
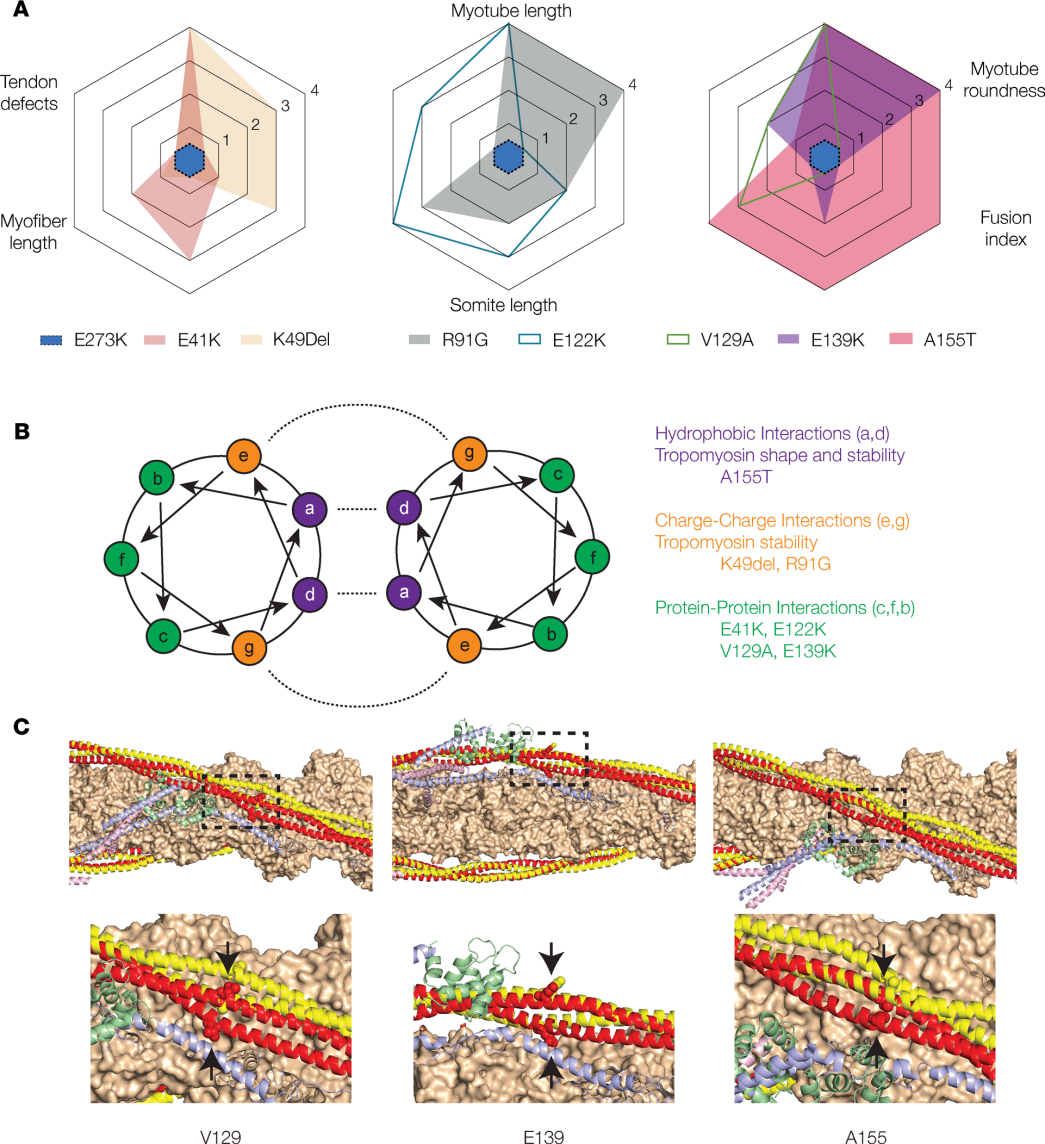
The impact of *TPM2* variants on musculoskeletal development correlates with intermolecular interactions. (**A**) A155T induced the most significant phenotypes among the *TPM2* variants tested. Radar plots of C2C12 cell and zebrafish phenotypes. Each assay was scored using statistical significance: 0 (not significant), 1 (*P* < 0.05), 2 (*P* < 0.01), 3 (*P* < 0.001), 4 (*P* < 0.0001). The score for each assay is graphed for each variant tested. E273K has a score of 0 for all assays. (**B**) Helical wheel model with described residues depicting the Tropomyosin dimer. Intermolecular interactions are shown with dashed lines. A155T occurs at a residue expected to promote hydrophobic interactions. (**C**) Thin filament structure involving potentially novel and recurring *TPM2* variants mapped onto the structure of the cardiac thin filament. Actin (orange), troponin I (blue), troponin C (green), and troponin T (pink) are shown from the low-calcium Cryo-EM structure (Protein Data Bank [PDB] 6KN7). Tropomyosin is shown in the low-calcium (red) and high-calcium (yellow) states, based on PDB 6KN7 and 6KN8, respectively. The mutated residues are shown as spheres (arrows).

A155T caused the most consistent and significant phenotypes of the variants we tested (Figure 11A), and patients with the A155T variant experienced a wider range of clinical symptoms than patients with other variants (Table 2). Patient III from this study was diagnosed with DA1 due to joint contractures but also showed proximal and distal muscle weakness. Five members of a family carrying the A155T variant were also diagnosed with DA1 due to ulnar bilateral joint contractures (48). An unrelated patient heterozygous for A155V presented with respiratory insufficiency that required multiple interventions, along with extreme muscle weakness in the limbs. Upon muscle biopsy, the patient was diagnosed with CM (57). Patients with A155 variants have therefore shown symptoms associated with both myopathies and arthrogryposes. In contrast, patients carrying the other variants we tested showed either myopathy-related muscle weakness (E41K, K49Del, E122K) or arthrogryposis-related contractures (R91G, V129A, E139K), but not both (Table 2). These results highlight the exciting possibility that our myogenesis and muscle function assays can predict the clinical severity of *TPM2* variants.

Mechanistically, the A155 residue is predicted to mediate intermolecular hydrophobic interactions that shape and stabilize the TPM2 dimer, and A155T is the only variant we tested that changes a residue involved in hydrophobic interactions within the Tropomyosin coiled-coil (Figure 11, B and C). K49Del and R91G affect residues involved in charged intermolecular interactions, and E41K, E122K, V129A, and E139K affect residues that likely mediate interactions between TPM2 and other thin filament proteins (Figure 11B). However, phenotypic severity in myogenic assays did not further subdivide between charged and protein-protein interactions. These observations suggest residues that mediate hydrophobic interactions are the most critical for TPM2 function during myogenesis, which is further supported by the fact that of the 25 known pathogenic variants, only A155T/V and Q218Del affect residues at sites of hydrophobic interactions. Of the 14 variants of uncertain significance in TPM2 that remain to be characterized, only Q276E is predicted to disrupt hydrophobic interactions. We hypothesize that variants affecting hydrophobic interactions are the least tolerated and therefore appear less in the patient population. This hypothesis predicts that Q276E will produce consistent and significant phenotypes in our myogenic assays.

## Methods

### Drosophila genetics

The *TPM2* and *Tm2* transgenic variants were constructed by PCR cloning the *TPM2* ORF (clone HsCD00368588, PlasmID) and the *Tm2* ORF (RE15528, Berkeley *Drosophila* Genome Project) into pEntr (Life Technologies), followed by recombination into destination vector TWG (Drosophila Genome Resource Center) to add a C-terminus GFP tag. TWG clones served as a template to generate variants by site-directed mutagenesis as described before (58). Tagged and untagged ORFs were PCR subcloned into pUASt.attB using EcoRI/XbaI. All constructs were targeted to the same attP site on chromosome 3L (65B2) using ΦC31 integrase and standard injection methods (Rainbow Transgenic Flies, Inc.). pUASt.attB constructs were fully sequenced prior to injection.

### Cell culture

*TPM2* mammalian expression constructs were generated by PCR subcloning variants from pUASt.attB into pCMV-IRES-eGFP (Addgene 78264) using XbaI/EcoRI. C2C12 cells (obtained from ATCC) were seeded in 6-well plates, grown in standard conditions to 60% confluence in growth medium (10% FBS in DMEM), and transfected with 1.5 μg of DNA per manufacturer’s specifications (Lipofectamine 3000, L3000015, Thermo Fisher Scientific). Empty pCMV-IRES-eGFP was used as a control. Growth media were changed to differentiation media (2% horse serum in DMEM) 24 hours after transfection; cells were differentiated for 7 days prior to fixation.

### Fish genetics and injections

Wild-type zebrafish were from line AB (obtained from the Washington University School of Medicine Zebrafish Consortium). *TPM2* variants were PCR subcloned from pUASt.attB into pCR2.1 (K202040, Thermo Fisher Scientific), capped RNAs were transcribed with a T7 mMessage mMachine kit (AM1344, Thermo Fisher Scientific), and embryos from natural spawning were injected with up to 600 pg RNA in phenol red (P0290, MilliporeSigma, 1:6). Injected embryos were maintained at 28.5°C in egg water and collected at 26 hours for histology or at 6 dpf for functional assays (feeding protocols began at 4 dpf). Multiple clutches were injected and pooled for each variant tested unless otherwise noted. Control injected larvae were collected to normalize each cohort.

### Immunohistochemistry, imaging, and image quantification

#### Drosophila.

Dechorionated embryos were fixed in 4% formaldehyde, devitellinated with heptane/methanol, and antibody stained as described before (58). Antibodies used were α-Mef2 (1:1000, gift from R. Cripps, San Diego State University, San Diego, California, USA; generated as described in ref. 59), α–myosin heavy chain (1:600, Abcam, catalog MAC147), α-GFP (1:600, Torrey Pines Biolabs, catalog TP-401), and α-βgal (1:100, Promega, catalog Z3781). HRP-conjugated secondary antibodies (goat anti-mouse Fluor 488 catalog 115-545-166, goat anti-rabbit Fluor 488 catalog 111-545-003, and goat anti-rat Fluor 594 catalog 112-585-167, all Jackson ImmunoResearch) in conjunction with the TSA system (Molecular Probes) were used to detect primary antibodies.

#### C2C12 cells.

Differentiated cells were fixed for 15 minutes in 4% paraformaldehyde (PFA), blocked in 5% NGS/PBS, and incubated overnight with α–alpha-actinin (catalog A7811, MilliporeSigma, 1:1000). Primary antibodies were visualized with an Alexa Fluor 594–conjugated secondary antibody (catalog 115-585-003, Jackson ImmunoResearch Laboratories); myonuclei were visualized with Hoechst (H3570, Thermo Fisher Scientific, 1:1000).

#### Zebrafish.

Hand-dechorionated larvae were fixed in 4% PFA for 1 hour and directly stained with Alexa Fluor 555–conjugated phalloidin (catalog A34055, Thermo Fisher Scientific, 1:200) for 2 hours at room temperature or blocked in 5% NGS/PBS-Triton-X 0.1% for 1 hour and incubated overnight with primary antibodies: THBS4 (Abcam, catalog ab211143, 1:100), myosin heavy chain (clone F59, Developmental Studies Hybridoma Bank, 1:50), and myosin light chain (clone F310, Developmental Studies Hybridoma Bank, 1:50). HRP-conjugated secondary antibodies in conjunction with the TSA system were used to detect primary antibodies.

#### Imaging.

Embryos and larvae were imaged with a Zeiss LSM800 confocal microscope; cells were imaged with an inverted Zeiss Axio Observer. Drosophila larvae were live-imaged in PBS-Tween 20 after 5-minute exposure to diethyl ether. For time-lapse imaging, dechorionated Stage 12 Drosophila embryos were mounted in halocarbon oil (MilliporeSigma) and scanned at 2-minute intervals. Control and treated samples were prepared and imaged in parallel where possible, and imaging parameters were maintained between treatment groups. Fluorescence intensity and cell morphology measurements were made with ImageJ software (NIH).

### Locomotion and startle response assays, hatching assays, Western blotting, quantitative real-time PCR, and protein modeling

See Supplemental Data 1.

### Clinical sequencing

All patients were recruited from St. Louis Children’s Hospital or Shriners Hospital St. Louis. The institutional review board approved this study and all patients and/or parents provided informed consent. Exome sequencing was performed as described before (60) on a cohort of patients with isolated clubfoot and distal arthrogryposis. Variants were validated by Sanger sequencing.

### Statistics

Statistical analyses were performed with GraphPad Prism 9 software, and significance was determined with the unpaired, 1-tailed Student’s *t* test; 1-way ANOVA; or nonparametric tests (for non-Gaussian distributions). Gaussian distribution fit curves were generated with Origin 2019 software. The box plots depict the 5th to 95th percentiles (whiskers), the upper and lower quartiles (boxes), and the median. Sample sizes are indicated in the figure legends, and *P* values less than 0.05 were considered significant. Data collection and data analyses were routinely performed by different authors to prevent potential bias. All individuals were included in data analysis.

#### Drosophila.

Muscle morphology and size were visualized by Tropomyosin-conjugated GFP in hemisegments A2–A8, using 6–10 Stage 16 embryos per genotype. For morphology, muscles were assigned a phenotype (normal, missing, misshapen, elongation defect, attachment site defect), reported as a frequency. Myoblast fusion was quantified by counting the number of lacZ^+^ myonuclei per hemisegment (A2–A8) in *rP298.nlacZ* embryos. Fusion index = (#lacZ nuclei experimental/#lacZ nuclei control) × 100.

#### Zebrafish.

Methods for measuring musculoskeletal parameters are shown in Figure 5 and largely reflect those reported previously (61). To control for day-to-day variability in embryo injections, muscle measurements were first normalized to the daily control and then reported as a percentage of control.

#### C2C12 cells.

Absolute myotube length was used for comparisons among treatment groups. Fusion index = (#nuclei in multinucleate myotubes/total nuclei ) × 100. A minimum of 10 fields were quantified per treatment for each parameter.

### Study approval

*Danio rerio* were maintained in accordance with approved institutional protocols under the supervision of the Institutional Animal Care and Use Committee of Washington University, which is fully accredited by the Association for Assessment and Accreditation of Laboratory Animal Care International. Drosophila work did not require committee oversight. Patient studies were performed under the approval of the Institutional Review Board of Washington University. Written informed consent was obtained from all participants or their guardians. Written informed consent was also provided for photos appearing in the manuscript.

## Author contributions

ANJ, CAG, MJG, JM, and SY conceived the study; ANJ, CAG, and SY developed methodology; SY, JM, GH, CAG, MJG, TO, MBD, and ANJ performed formal analysis; SY, JM, GH, and ANJ investigated; CAG and ANJ provided resources; SY, JM, and ANJ curated data; ANJ, CAG, and MJG wrote the original draft; SY and ANJ visualized data; SY, JM, and ANJ supervised the study; ANJ performed project administration; CAG, MJG, and ANJ acquired funding.

## Supplementary Material

Supplemental data

Supplemental video 1

Supplemental video 2

Supplemental video 3

## Figures and Tables

**Table 1:**
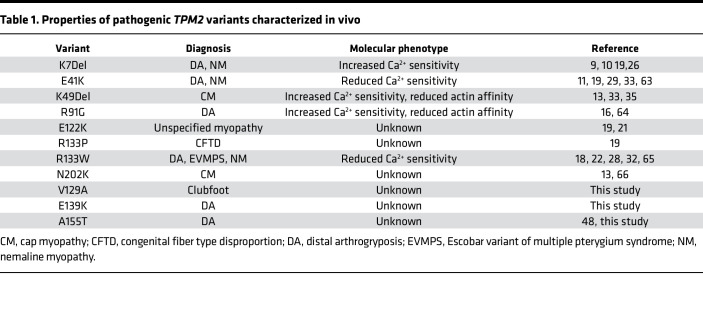
Properties of pathogenic *TPM2* variants characterized in vivo

**Table 2:**
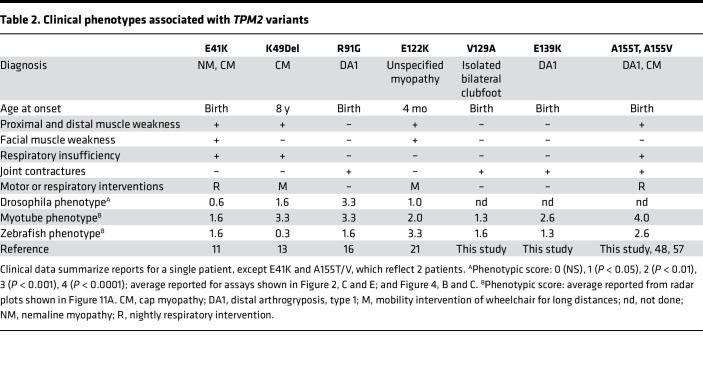
Clinical phenotypes associated with *TPM2* variants

**Table 3:**
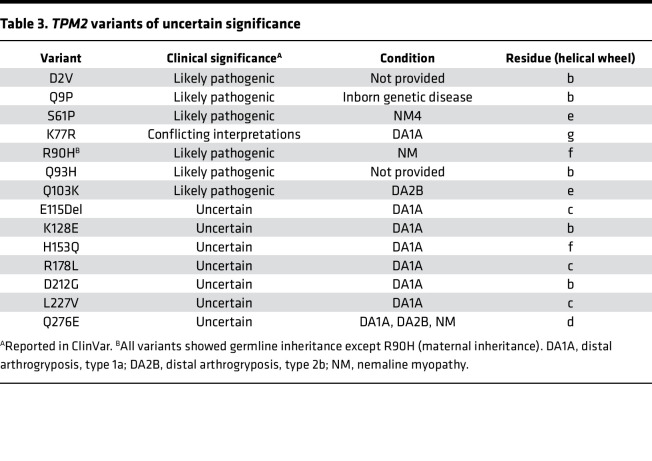
*TPM2* variants of uncertain significance

## Version 1 05/17/2022

In-Press Preview

## Version 2 06/22/2022

Electronic publication
