# Supplementary material for: A pathogenic mechanism associated with myopathies and structural birth defects involves TPM2-directed myogenesis
Source: JCI Insight. 2022 Jun 22;7(12):e152466. doi: 10.1172/jci.insight.152466 (PMC9309062; doi:10.1172/jci.insight.152466)
Supplement: Supplemental data [file jciinsight-7-152466-s025.pdf]

Supplemental Data

Methods

Drosophila genetics

Additional stocks used in this study were *P{GMR40D04-GAL4attP2* (*slou.Gal4*), *P{GMR57C12-GAL4}attP2* (*nau.Gal4*), $Tm2^{D8\text{-}261}$ [8], *P(lacZ-kirre*^{rP298}*)* [59], and *P(PTT-GC)*$Tm2^{ZCL2456}$ [60]. *Cyo, P(wg.lacZ), Cyo, P(twi.Gal4), P(UAS.GFP), TM3, P(ftz.lacZ),* and *TM3, P(twi.Gal4), P(UAS.GFP)* balancers were used to identify homozygous embryos. Fly stocks were obtained from the Bloomington Stock Center unless otherwise referenced.

Locomotion and Startle Response Assays

Drosophila L3 larva locomotion assays were performed as described [42]. For zebrafish larva, the Noldus DanioVision and EthoVision software were used to record and quantify larval movement at 6dpf as described [45]. Larva were loaded into the DanioVision in 24-well cell culture plates with egg water at random, and acclimated in the DanioVision box for 5-10 min. The culture plate was automatically tapped after acclimation, and the startle response was recorded for 3s; EthoVision software tracked and recorded fish movement, and reported escape velocity and distance traveled. Statistical analyses were performed only between control and experimental groups assayed on the same day.

Hatching Assays

0-24hr old embryos were collected on grape agar plates, dechorionated, and genotyped using GFP expression from the *TM3, P(twi.Gal4), P(UAS.GFP)* balancer. Genotyped embryos were transferred to a grape agar plate, incubated for 24hr at 25°C, and then scored for hatching. At least two collections were completed per condition.

Western blotting

C2C12 cells. Cells were lysed with 600μl IP buffer (20 mM Hepes, pH=7.4, 150 nM NaCl, 1% NP40, 1.5 mM $MgCl_2$, 2 mM EGTA, 10 mM NaF, 1 mM Na_3VO_4, 1X proteinase inhibitor), incubated on ice for 30 min, and centrifuged at 12000Xg for 15min. Supernatants were collected, and Western blots were performed by standard method using precast gels (#456-1096, BioRad), and imaged with the ChemiDoc XRS+ system (BioRad).

zebrafish. Embryos and larvae were collected and homogenized with 600μl IP buffer, and centrifuged at 12000Xg for 15min to remove large debris. Supernatants were blotted as described above.

Quantitative real time PCR

Total RNA was extracted with RNeasy mini kit (74104, Qiagen), and quantified (Nanodrop 2000). cDNA was prepared by reverse transcription with M-MLV Reverse Transcriptase (28025013, Thermo) using 2000ng RNA. PowerUp Sybr Green Master Mix (A25742, Thermo) and ABI StepOne system (Applied Biosystems) were used for quantitative RT-PCR. Quantification was normalized to *RpL32*. Primers used were:

TPM2-F-5`-???-3`

TPM2-R-5`-???-3`

Rp32- F-5`-ATGCTAAGCTGTCGCACAAATG-3`

Rp32- R-5`-GTTCGATCCGATACCGATGT-3`

Protein modeling

The position of residues in the coiled-coil structure was calculated using [62]. To visualize the position of tropomyosin along the thin filament, we looked at analogous residues in the structures of the cardiac thin filament solved using cryoelectron microscopy in the presence (PDB ID: 6KN8) and absence (PDB ID: 6KN7) of calcium [63]. Structures were visualized in PyMol.

Movie Captions

Movie 1. K49Del expressing myotubes fail to elongate. LO1 myotubes are pseudocolored blue (WT) or red (K49Del). The K49Del expressing LO1 myotube initiated elongation, but then retracted and failed to reach the muscle attachment site.

Movie 2. K49Del expressing myotubes develop a third leading edge. LO1 myotubes are pseudocolored blue (WT) or red (K49Del). The K49Del expressing LO1 myotube initiated elongation with two leading edges, but then developed a third leading edge that appeared to attach to a tendon cell by the end of the movie. Notice a motile cell pinched off of the mutant LO1 and left the filed of view (psuedocolored yellow).

Movie 3. E122K expressing myotubes elongate incorrectly. LO1 myotubes are pseudocolored blue (WT) or red (E122K). The E122K expressing LO1 myotube elongated toward an incorrect muscle attachment site.
